# Comprehensive evaluation framework for synthetic tabular data in health: fidelity, utility and privacy analysis of generative models with and without privacy guarantees

**DOI:** 10.3389/fdgth.2025.1576290

**Published:** 2025-04-24

**Authors:** Mikel Hernandez, Pablo A. Osorio-Marulanda, Mikel Catalina, Lorea Loinaz, Gorka Epelde, Naiara Aginako

**Affiliations:** ^1^ Digital Health and Biomedical Technologies, Vicomtech Foundation, Basque Research and Technology Alliance (BRTA), Donostia-San Sebastián, Spain; ^2^ Computer Science and Artificial Intelligence Department, Computer Science Faculty, University of the Basque Country (UPV/EHU), Donostia-San Sebastián, Spain; ^3^ eHealth Group, Biogipuzkoa Health Research Institute, Donostia-San Sebastián, Spain; ^4^ School of Applied Sciences and Engineering, Universidad EAFIT, Medellín, Colombia

**Keywords:** synthetic data generation, generative models, synthetic data fidelity, synthetic data utility, privacy risk measureattacks, synthetic data evaluation, differential privacy, synthetic data privacy

## Abstract

The generation of synthetic tabular data has emerged as a key privacy-enhancing technology to address challenges in data sharing, particularly in healthcare, where sensitive attributes can compromise patient privacy. Despite significant progress, balancing fidelity, utility, and privacy in complex medical datasets remains a substantial challenge. This paper introduces a comprehensive and holistic evaluation framework for synthetic tabular data, consolidating metrics and privacy risk measures across three key categories (fidelity, utility and privacy) and incorporating a fidelity-utility tradeoff metric. The framework was applied to three open-source medical datasets to evaluate synthetic tabular data generated by five generative models, both with and without differential privacy. Results showed that simpler models generally achieved better fidelity and utility, while more complex models provided lower privacy risks. The addition of differential privacy enhanced privacy preservation but often reduced fidelity and utility, highlighting the complexity of balancing fidelity, utility and privacy in synthetic data generation for medical datasets. Despite its contributions, this study acknowledges limitations, such as the lack of evaluation metrics neither privacy risk measures for required model training time and resource usage, reliance on default model parameters, and the assessment of models that incorporates differential privacy with only a single privacy budget. Future work should explore parameter optimization, alternative privacy mechanisms, broader applications of the framework to diverse datasets and domains, and collaborations with clinicians for clinical utility evaluation. This study provides a foundation for improving synthetic tabular data evaluation and advancing privacy-preserving data sharing in healthcare.

## 1 Introduction

Recent advancements in digital health have enabled the design and development of sophisticated data analysis technologies that enhance diagnostics, personalised treatment plans, and predictive healthcare solutions among other applications (1). However, fully leveraging these advances requires access to large amounts of data to ensure the reliability of data analysis paradigms and foster a data-driven approach to healthcare (2). Much of the data collected for these purposes contains sensitive information that may identify individuals or reveal personal details about them, raising significant privacy concerns. As a result, strict regulations and ethical standards, such as the General Data Protection Regulation (GDPR) (3) or Health Insurance Portability and Accountability (HIPAA) (4), often restrict or delay the external sharing of this data.

Synthetic tabular data generation (STDG) through generative models has emerged as a key privacy-enhancing technology (PET) in digital health (5) to address these challenges. This technology uses generative models to create realistic, non-identifiable data that preserves the essential statistical properties and relationship of the real data while mitigating privacy risks. This enables researchers to share data securely, fostering innovation in applications such as decision support systems, disease prediction, and other healthcare solutions (6, 7).

Despite the promises of STDG, its application in the health domain presents several challenges. Medical datasets are complex and have diverse attributes that need to be accurately modelled to maintain data fidelity (how closely the synthetic data resembles the original), utility (its usability for analytical tasks) and privacy (protection against sensitive information leaks). Balancing these three dimensions is crucial when generating synthetic tabular data that can be a proxy for real tabular data, especially when sharing it with external researchers and entities, which poses a gap on synthetic tabular data evaluation benchmarks and frameworks (8).

Several attempts have been made to propose evaluation frameworks covering different categories. Specifically, Hittmeir et al. (9), Rankin et al. (10), Dankar et al. (11) and El Emam et al. (12), have primarily focused on evaluating the utility of synthetic data. On the other hand, works by Hernandez et al. (13), Lautrup et al. (14), Livieris et al. (15), Höllig et al. (16), Cheng-Hsin et al. (17) and Vibeke et al. (18) also considered privacy and fidelity, providing usage examples to benchmark STDG models. Beyond these proposals, other evaluation categories have also been explored in the literature. For example, Vibeke et al. (18) included fairness, fingerprint and computational complexity, while Stenger et al. (19) proposed an evaluation taxonomy including diversity and generalisation. However, these categories are more related to the evaluation of the generative models themselves rather than the generated synthetic data.

While different synthetic data evaluation frameworks exist, all the listed studies lack a clear consensus on which metrics and privacy risk measures should be used in each evaluation category and acceptable ranges for them, making it difficult to standardise synthetic data evaluation. Furthermore, a recent scoping review by Kaabachi et al. (20) highlights the extensive and varied research on synthetic data evaluation in the health domain, with no clear agreement on the most suitable metrics and methods for different use cases. To this end, Stenger et al. (19) proposed a standardised synthetic data evaluation framework with a broad taxonomy of quality categories (authenticity, generalization, interpretability, etc.) that defines a common language and evaluation criteria but focused specifically on synthetic time series data. Although it is conceptually aligned with the goal of this paper, their proposed categories are closely tied to the temporal structure of time series and are not directly applicable to tabular data. Despite these efforts, gaps remain in providing a comprehensive and adaptable framework capable of assessing and comparing the performance of different STDG models in a standardised manner while ensuring fidelity, utility, and privacy.

In this context, we propose a robust and flexible evaluation framework that rigorously assesses and compares synthetic tabular data generated by various STDG models. Building upon previous work by Hernandez et al. (13), our framework consolidates a minimal yet robust set of metrics and privacy risk measures carefully chosen to capture all critical features of the generated synthetic tabular data while avoiding redundancy across the three key evaluation dimensions (fidelity, utility, and privacy). Additionally, the metric introduced by Galloni et al. (21) to quantify the tradeoff between fidelity and utility under different privacy constraints is integrated into our framework to enable a holistic evaluation of the synthetic tabular data.

To demonstrate the effectiveness of our evaluation framework, we apply it to three open-source medical datasets, comparing synthetic data generated by different STDG models, both with and without differential privacy (DP). The results of this validation allow us to assess whether incorporating DP to STDG models impact significantly the fidelity, utility and privacy of the generated synthetic tabular data, and to determine the most suitable STDG model for different use cases (i.e., data augmentation or privacy preservation). By providing a detailed evaluation framework, this paper aims to help researchers, healthcare organisations, and policymakers make informed decisions about using synthetic data for data-driven healthcare solutions while ensuring privacy protection and maintaining the utility of the data.

## 2 Materials and methods

### 2.1 Synthetic tabular data evaluation framework

This subsection provides a detailed overview of the metrics and privacy risk measures included in the proposed synthetic tabular data evaluation framework for each evaluation category (fidelity, utility, privacy and tradeoff between fidelity and utility) that are summarised in Table 1. These evaluation categories were selected because they are widely accepted in the literature as core dimensions for evaluating synthetic tabular data, focusing on how well the synthetic data resembles the real data (fidelity), how useful it is for downstream applications (utility), and how effectively it protects sensitive information (privacy) (6). While other categories such as efficiency, generalization, or interpretability have been analysed in the literature, they are more related to the evaluation of the STDG themselves rather than the generated synthetic tabular data.

**Table 1:** Summary of the synthetic tabular data evaluation metrics and privacy risk measures included in the framework.

Category	Metric or measure	Description	Range	Best
Fidelity	Hellinger distance	Distance metric that quantifies the similarity between distributions of real and synthetic attributes.	[0,1]	≈0
	Pairwise correlation difference (PCD)	Mean difference between real and synthetic data pairwise correlations.	[0,1]	≈0
	$R^{2}$ of DD-plot	Real data depth adjustment to the depth of the synthetic data.	[0,1]	≈1
	AUC-ROC	Ability of a classifier to distinguish between real and synthetic samples.	[0,1]	≈0.5
Utility	Classification metrics differences	Absolute difference in classification metrics (accuracy, precision, recall, f1-score) between predictive models trained on real data and on synthetic data apart for categorical attributes.	[0,1]	≈0
	Regression metrics differences	Absolute difference in regression metrics (mae, mse, rmse and $r^{2}$) between predictive models trained on real data and on synthetic data apart for numerical attributes.	[0,1]	≈0
Privacy risk measures	Univariable singlingout	Success rate in finding out that a record with a specific attribute exists in the real data.	[0,1]	≈0
	Multivariable singlingout	Success rate in finding out that a record with a specific set of attributes exists in the real data.	[0,1]	≈0
	Linkability	Success rate in linking an existing record in the real and synthetic data corresponding to the same record.	[0,1]	≈0
	Membership inference	Success rate in linking an existing record in the real data to a set of records in the synthetic data.	[0,1]	≈0
	Attribute inference	Success rate in linking a set of attributes of the synthetic data that correspond to the same set of attributes of the real data.	[0,1]	≈0
Tradeoff	$G_{\epsilon }$	Tradeoff between fidelity and utility of synthetic data.	[0,1]	≈0

#### 2.1.1 Fidelity

Metrics within the fidelity category assess the ability of synthetic tabular data to accurately represent real tabular data in terms of distributions, correlations, adjustments, and distinguishability.

##### 2.1.1.1 Hellinger distance

The Hellinger distance quantifies the similarity between two probability distributions, serving as a bounded metric in the space of probability distributions over a given probability space. This distance metric was chosen mainly for its robustness across both numerical and categorical attributes, its bounded nature (0 to 1), and its focus on probability distributions. Unlike other distance metrics, such as Wasserstein or Kullback-Leibler, the Hellinger distance offers a normalized and interpretable measure of marginal distributions and is less sensitive to outliers or extreme values. This makes this distance metric particularly suitable and robust for comparing real and synthetic data univariate distributions in mixed-type tabular data.

For two probability distributions (P and Q), the Hellinger distance is directly related to the Euclidean norm of the difference between the square root vectors, as shown in Equation 1. Here, P represents the attribute of the real tabular data, and Q represents the attribute of the synthetic tabular data. This distance metric is computed for each attribute between real and synthetic tabular data to assess the similarity in probability distributions.

(1) $$H(P,\;Q)=\frac{1}{\sqrt{2}}\sqrt{\sum_{i=1}^{k}{\left(\sqrt{\;p_{i}}-\sqrt{q_{i}}\right)}^{2}}$$

If the distance is 0 (the minimum possible value), the two probability distributions (real and synthetic) are identical. If the distance is 1 (the maximum possible value), the distributions of the real and synthetic attributes are as opposite as possible. Therefore, the lower and closer to 0 the Hellinger distance, the more similar the distributions of the real and synthetic attributes. To summarize the results for each variable in a dataset, the average of all the Hellinger distances across all attributes is calculated.

##### 2.1.1.2 Pairwise correlation difference

The Pairwise Correlation Difference (PCD) is a well-established and widely used metric to quantify the difference between the pairwise correlations in real and synthetic tabular data. It captures how well the synthetic data preserves the correlations among variables in the real data in a simple and interpretable way, which makes it a standard choice for synthetic data fidelity evaluation.

This measure is computed by extracting the upper triangular part (excluding the diagonal) of the correlation matrix from real and synthetic data, resulting in a vector of n unique correlation values, where n is the number of unique variable pairs. Being $\text{Corr}(X_{\text{real}}{)}_{i}$ the correlation between the i-th variable pair in real data and $\text{Corr}(X_{\text{synth}}{)}_{i}$ the correlation between the i-th variable pair in synthetic data, the PCD is computed as shown in Equation 2, where the absolute differences between all pairwise correlations are averaged. A low value (close to 0) indicates that the correlations in the synthetic data do not differ much from those in the real data. In contrast, a higher value (close to 1) implies that the correlations in the synthetic tabular data are strongly different from the correlations in the real data. To assess whether there is a statistical difference between these correlation differences, this metric is complemented by a Student’s t-test with a significance level of 5% to determine if the differences in correlations are statistically significant.

(2) $$PCD(X_{\text{real}},\;X_{\text{synth}})=\frac{1}{n}\sum_{i=1}^{n}\left|\text{Corr}(X_{\text{real}}{)}_{i}-\text{Corr}(X_{\text{synth}}{)}_{i}\right|$$

For the calculation of the correlation matrices for both the real and synthetic datasets, we propose using the correlation constant $\phi_{k}$ introduced by Baak et al. (22). This constant works consistently across categorical and numerical variables, captures non-linear relationships between variables, and defaults to the pearson correlation coefficient in the case of bivariate normal input distributions. Therefore, this correlation constant allows computing the correlation matrix of variables with mixed types of variables.

##### 2.1.1.3 Coefficient of determination of the Depth vs. Depth plot

The Depth vs. Depth Plot (DD-Plot) is a non-parametric method introduced by Regina et al. in 1999 (23) to plot the depth values of combined samples under two corresponding distributions. This method was selected because, unlike marginal distribution measures, it evaluates the multivariate distributional similarity between real and synthetic data, providing a more holistic view of the data structure, making it a robust tool for comparing high-dimensional tabular datasets.

Following the approach followed by Restrepo et al. (24), the plot aims to compare the depth of measurements obtained from synthetic tabular data with those from real tabular data, representing the real data depths on the *X*-axis and the synthetic data depths on the *Y*-axis. If both distributions are identical, the plot should show a set of points aligned along the line y=x. Points located close to this line suggest a high agreement between the synthetic and real tabular data, while deviations from this line indicate discrepancies.

The coefficient of determination ($R^{2}$) is proposed to provide an analytical value to this plot. This metric indicates the proportion of variance in the dependent variable (synthetic depths) that can be predicted from the independent variable (real depths). It quantifies the degree of agreement between the real and synthetic dimensions, with 1 pointing that the synthetic samples come from the same multivariate distribution as the real samples, and 0 representing a lack of linear relationship between multivariate distributions of synthetic and real samples. This combination of visual analysis with an associated analyticial value enhances the interpretability of multivariate fidelity in synthetic data.

##### 2.1.1.4 Area under the receiving operating characteristic curve

The Area Under the Receiver Operating Characteristic Curve (AUC-ROC) measures the ability of a binary classifier to distinguish between different classes across various threshold settings, making it commonly used to evaluate how distinguishable synthetic samples are from real ones. A value around 0.5 indicates that the classifier performs no better than random guessing, suggesting high similarity between real and synthetic samples. On the other hand, a value significantly higher than 0.5, with a possible maximum of 1, implies that the classifier is effective in distinguishing between real and synthetic samples pointing to lower fidelity. Although, the AUC-ROC has limitations in certain settings, it is a robust and widely accepted metric to effectively capture the distinguishability between real and synthetic samples.

To compute the AUC-ROC and analyze the distinguishability of synthetic samples from real samples, real and synthetic samples are combined into a single dataset, and a Random Forest classifier is trained with a maximum depth of 3 decision trees, 1,000 estimators, and the out-of-bag (OOB) score function enabled with AUC-ROC. This classifier was selected due to its robustness, its ability to handle mixed-type tabular data, and its minimal reliance on hyperparameter tuning. The AUC-ROC value is then obtained by averaging the OOB scores across all trees, which offers an internal cross-validation mechanism by computing scores on test samples that were not used in training the corresponding decision trees. This method allows for a controlled and reproducible comparison of real and synthetic data without requiring a separate validation set.

#### 2.1.2 Utility

To evaluate the utility of synthetic tabular data, its performance in real-world data analysis tasks was assessed by comparing the results to those obtained using real tabular data, following the widely used methodology suggested by Hernandez et al. (13) and Rankin et al. (10). The goal is to determine whether synthetic tabular data can effectively replace real data for training machine learning models. Specifically, we aim to verify if models trained with synthetic data produce results comparable to those trained with real data when validated on the same real test samples.

The utility evaluation follows two standard training-testing scenarios with several machine learning models: (1) training on real tabular data, tested on a real test sample (TRTR), and (2) training on synthetic tabular data, tested on the same real test sample (TSTR). The performance of TRTR models serves as the baseline, and the mean absolute difference between TRTR and TSTR models is calculated. A smaller difference (closer to 0) indicates better utility of the synthetic tabular data. In contrast to previous works, which often assess utility based on a single (typically categorical) target variable, we train predictive models for all variables in each dataset This provides a more robust and comprehensive view of the utility of synthetic data across diverse tasks.

For this analysis, classification models are applied for categorical attributes, and regression models are used for numerical attributes. To ensure broad applicability, five widely adopted ML models were considered: Random Forest, K-Nearest Neighbors, Decision Trees, Support Vector Machines, and Multilayer Perceptron. Performance differences of these models are evaluated using standard metrics: accuracy, precision, recall, and F1-score for classification tasks, and MAE, MSE, RMSE, and $R^{2}$ for regression tasks. To summarise the results, we compute the mean difference between TSTR and TRTR models for each metric, along with the associated p-value obtained through a t-test comparing the means of the two samples. If the p-value exceeds the significance level of 0.05, we conclude that there is no statistically significant difference between the TRTR and TSTR models concluding that the synthetic data provides similar utility to the real data.

#### 2.1.3 Fidelity-utility tradeoff

The G metric proposed by Galloni et al. (21) is added to the framework to provide a single, interpretable value that captures the ability of synthetic data to preserve utility while maintaining fidelity to the real data. This is particularly useful when comparing different generative models or parameter configurations, as it condenses two critical aspects of synthetic tabular data (fidelity and utility) into a single score. The metric assesses the balance between fidelity and utility in synthetic tabular datasets under different privacy constraints. It evaluates synthetic tabular data in two key dimensions; statistical fidelity and data utility. This value combines these two aspects, weighting equally the PCD and the TSTR-TRTR metrics differences for different machine learning tasks to provide a comprehensive evaluation. A lower G value (closer to 0) indicates a better balance between preserving utility and maintaining fidelity, while higher values suggest a weaker tradeoff. This metric is especially relevant when evaluating the impact of DP, as it allows to analyze how applying DP to the STDG model with different noise addition (ϵ) settings can affect this tradeoff, creating the $G_{\epsilon }$ metric.

#### 2.1.4 Privacy risk measures

Privacy risk measures estimate the performance of an external agent attempting to extract sensitive information from a real dataset if they gain access to the synthetic dataset. These privacy simulations measure the effectiveness of the synthetic tabular data in preserving data privacy.

The selection of privacy risk measures follows the framework proposed by Giomi et al. (25), which identifies singling out, linakbility and inference risks as key indicators of factual anonymization according to data protection regulations. These privacy risk measures are therefore widely accepted as meaningful for evaluating the privacy of synthetic data. Additionally, the membership inference risk was included, which is one of the most commonly used privacy attack, according to the synthetic data review developed by Osorio-Marulanda et al. (5). This attack complements the others by addressing the specific risk of attribute disclosure. For the proposed evaluation framework, the success probability of an adversary executing the following privacy attacks was simulated and quantified:

• Singling out: This attack occurs when a unique data record can be identified based on a distinct combination of attributes within the real tabular data. The attack is performed for each attribute individually (univariate singling out) and in conjunction with all attributes (multivariable singling out). The objective of these attacks is to identify a specific record based on its attribute combination, even when other records in the real dataset have similar attributes.
• Linkability: This attack arises when attributes from two or more records, either within the same dataset or across different datasets, can be linked to the same individual or group. The attack is deemed successful if the known attributes and a synthetic dataset allow linking information back to the real dataset, revealing the identity or sensitive details of a individual or a group of individuals in the real dataset.
• Membership inference: This attack involves associating a record from the real dataset with a set of records in the synthetic dataset. It identifies the closest synthetic record to a given real record and calculates the distance between them. The attack succeeds if the distance falls below a specific threshold, enabling the adversary to infer whether a particular record is present in the original dataset.
• Attribute inference: This attack assumes that the adversary has access to a subset of attributes for a synthetic sample. The attacker infers unknown attribute values from the closest synthetic record, comparing the guessed values to the actual values. The attack is successful if the inferred values are sufficiently accurate (for numerical or continuous attributes) or match the correct category. This allows the attacker to deduce sensitive attributes from synthetic records.

For each of these attacks, the success rate is computed using the anonymeter framework created by Giomi et al. (25), except the membership inference which was implemented following a similar approach as the other attacks of this framework and based on the proposal done by Hernandez et al. (13). A test sample of real tabular data was used as the control data for all attacks. A lower success rate (close to 0) indicates that the synthetic data effectively preserves the privacy of the real dataset and does not contain sensible or personal information, while a higher success rate (close to 1) points to the synthetic data containing sensible or personal information.

### 2.2 Framework application

#### 2.2.1 Datasets

Three open-source health-related datasets were selected, covering different pathologies, data sizes, and types, as baselines for generating synthetic tabular data. These datasets were subsequently used to apply and validate the proposed evaluation framework. The datasets are described as follows:

• Acute myeloid leukemia dataset: Created by Papaemmanuil et al. in 2016 (26), this dataset contains metadata and medical biomarkers from 1,540 patients diagnosed with Acute Myeloid Leukemia (AML). These patients participated in three prospective medical trials conducted by the German-Austrian AML Study Group (AMLSG). The dataset includes a total of 12 variables: 6 numerical and 6 categorical.
• Brain stroke dataset: Developed by Tianyu et al. in 2019 (27), this dataset comprises 38,962 records, consisting of patient metadata and risk factors for predicting the likelihood of a brain stroke. It includes 11 attributes: 3 numerical and 8 categorical.
• Cardiovascular disease dataset: Published by Svetlana Ulianova in 2019 (28), this dataset contains 70,000 records with metadata and risk factors to predict the occurrence of cardiovascular disease. The dataset includes a total of 13 attributes: 6 numerical and 7 categorical.

Each dataset was pre-processed, deleting missing values and performing a data split into two subsets. 80% of the samples were used for training and evaluating the STDG models, while the remaining 20% were reserved as a test set. The test set was used for two purposes: evaluating machine learning models trained with synthetic tabular data and serving as a control set for the privacy attacks simulations presented in Section 2.1.4. Table 2 summarises the final number of samples and attributes for each dataset after pre-processing. A more detailed description of each dataset’s attributes and descriptive statistics of them is provided in Tables S1–S3 of the Supplementary Material.

**Table 2:** Characteristics of the selected health-related datasets.

Dataset name	Num. attributes	Cat. attributes	Train samples	Test samples
Acute myeloid leukemia dataset (26)	6	6	1,232	308
Brain stroke dataset (27)	3	8	31,169	7,793
Cardiovascular disease dataset (28)	6	7	56,000	14,000

#### 2.2.2 Synthetic tabular data generation models

To generate synthetic tabular data for the tabular medical datasets presented previously in Section 2.2.1 we used diverse state-of-the-art STDG models, including copulas, generative adversarial networks (GAN), variational autoencoders (VAE) and diffusion models (DM). These models were chosen to include different typologies of generative models:

• Nonparametric copula (NPC): This STDG model, proposed by Restrepo et al. (24) and adapted by Osorio-Marulando et al. (29) to make it compatible with multi-type variables and DP, generates synthetic tabular data by modelling the dependencies between attributes through an empirical copula and the marginal distributions of the real tabular data. It uses random uniform data that is transformed using these distributions to generate synthetic tabular data.
• Gaussian copula (GC): This STDG model is also based on real tabular data statistical modelling. Using a Gaussian copula to combine marginal probabilities estimated using univariate distributions, the model developed by Patki et al. (30) implements a multivariate distribution to generate synthetic tabular data. It was successfully used for medical datasets by Goncalves et al. (31), Yale et al. (32) and Hernandez et al. (13) and is available in a Github repository (33).
• Conditional tabular generative adversarial network (CTGAN): A GAN based model proposed by Xu et al. (34) designed and adapted by Patki et al. (30) for synthetic tabular data generation. As usually in GAN architectures, the model comprises a generator that generates synthetic data and a discriminator that distinguishes the synthetic and real samples. After various epochs, these two neural networks are trained adversarially to generate high-quality synthetic tabular data. This model was successfully used for medical datasets by Goncalves et al. (31), Yale et al. (32) and Hernandez et al. (13) and is available in a Github repository (33).
• Tabular variational autoencoder (TVAE): A VAE based model proposed by Xu et al. (34) designed and adapted by Patki et al. (30) for synthetic tabular data generation. The model comprises a neuronal network with a variational encoder and decoder. The encoder encodes the data to a known distribution while the decoder decodes it, generating the synthetic samples. This model was successfully used for medical datasets by Goncalves et al. (31) and Yale et al. and is available in a Github repository (33).
• Table-diffusion (TabDif): This STDG introduced by Gianluca et al. (35) is based on a diffusion model (DM). It iteratively removes noise added during a diffusion process to generate new synthetic samples. After this denoising process, the model learns to generate new synthetic samples from pure noise. This model is available in a Github repository (36)

To ensure privacy in these types of STDG models, differential privacy (DP) is commonly used. This mechanism was formally defined by Cynthia Dwork in 2008 (37) and introduces carefully calibrated noise into the data generation process to ensure that synthetic data does not reveal sensitive information about individuals in the real dataset. This noise is usually controlled by the privacy budget (ϵ), which balances privacy and utility: lower ϵ values ensure stronger privacy with more noise, while higher ϵ values provide better utility with weaker privacy.

By applying DP to the previously defined STDG models, we trained each STDG model with and without DP, totalling ten models, divided into pairs according to their typology: NPC vs. DP-NPC, GM vs. DP-GM, CTGAN vs. DP-CTGAN, TVAE vs. DP-TVAE, and TabDif vs. DP-TabDif. All these models were trained with default parameters and using an ϵ value of 1 for the STDG models that incorporate DP. Thus, the proposed metric to evaluate the tradeoff between fidelity and utility (G) is only applied with a unique ϵ value, being G for the STDG models without DP and $G_{1}$ for the STDG models with DP.

#### 2.2.3 Experimental procedure

**Figure 1:**
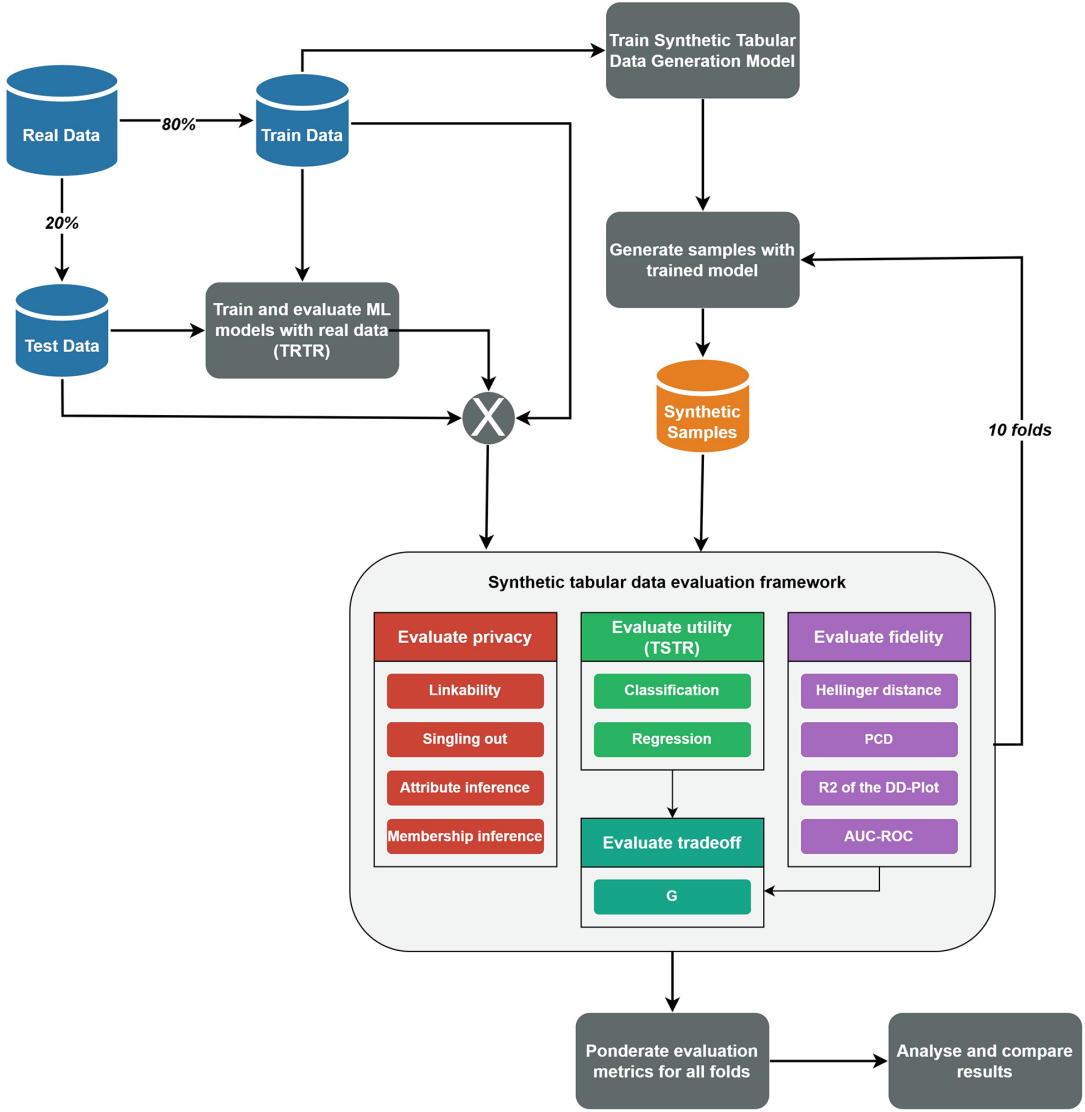
Flow diagram of the used procedure to apply the synthetic tabular data evaluation framework

The experimental procedure for applying the synthetic tabular data evaluation framework is illustrated in Figure 1. This figure outlines the steps followed for each combination of dataset and STDG model. The process begins by randomly splitting the real dataset into training (80%) and test (20%) sets using a fixed random seed to ensure reproducibility. This split was performed only once and used consistently throughout the evaluation to avoid retraining the synthetic data generation models multiple times and reduce computational costs. Next, the TRTR methodology is applied using the classification and regression models described in Section 2.1.2. These initial results serve as the baseline for evaluating the utility of the synthetic tabular data. After that, the STDG model is trained, and a synthetic dataset of the same size as the training set is generated. The evaluation framework is then applied to this synthetic dataset and repeated for 10 folds, simulating a cross-validation approach. Finally, the results from each fold are aggregated by calculating the best, worst, and average values for each metric and privacy risk measure. All this flow was executed using the Python programming language, and the open-source MLflow platform (38) was used to track the trained models and store the results in an organised manner for easy access.

### 2.3 Framework validation

To validate the effectiveness of the proposed synthetic tabular data evaluation framework in comparing and evaluating different STDG models, we applied the experimental procedure described in Section 2.2.3 to each dataset and STDG model combination outlined in Sections 2.2.1, 2.2.2. The primary goal of this validation is to assess the ability of the evaluation framework to two key challenges of STDG in the health domain:

1. Analyse the impact of incorporating the DP mechanism into different STDG model types on the fidelity, utility and privacy of the generated synthetic tabular data. Specifically, the statistically significant differences in each evaluation category of the synthetic tabular data generated with STDG models with and without DP were analysed.
2. Analyse the suitability of different STDG model types for medical tabular datasets for different use cases, specifically, data augmentation and privacy preservation. To this end, we selected the best and worst-performing STDG models for each dataset based on their performance across the evaluation categories.

To address these aspects, the results obtained from applying the evaluation framework were analysed. The mean values for each metric and privacy risk measure across all evaluated models and folds were recorded. Additionally, a paired t-test was performed to compute p-values for each metric and privacy risk measure, comparing the results of STDG models with and without DP. This statistical analysis enabled to determine whether incorporating DP leads to significant differences in the quality of the synthetic data generated by the same STDG model. Finally, the metric and privacy risk measure results across different model types were compared.

## 3 Results

This section presents the results obtained by applying the proposed synthetic tabular data evaluation framework using the application methodology detailed in Section 2.2. For each of the three datasets, we summarise the resulting evaluation metrics and privacy risk measures and briefly discuss the impact of adding DP to the STDG models and the identification of the best-performing STDG model. A more extended description of the results is provided in the Supplementary Material.

### 3.1 Acute myeolid leukemia dataset

#### 3.1.1 Fidelity and tradeoff

The fidelity and tradeoff metrics for the Acute Myeolid Leukemia dataset as summarised in Table 3, showing the mean values across the 10 evaluation folds. Figure S5 of the Supplementary Material provides a visual representation of the variability and significance of these metrics across the STDG models.

**Table 3:** Fidelity and tradeoff results summary for the acute myeloid leukemia dataset.

Model	Hellinger distance	PCD	$R^{2}$ of DD-plot	AUC-ROC	Tradeoff (G)
NPC	0.2193	0.0624	0.9975	0.6568	0.0211
DP-NPC	0.2539	0.0627	0.9114	0.7418	0.0498
*p-value*	$<0.05^{*}$	>0.05	$<0.05^{*}$	$<0.05^{*}$	$<0.05^{*}$
GC	0.2124	0.0668	0.9301	0.7504	0.0393
DP-GC	0.3006	0.1562	0.9401	0.8809	0.1028
*p-value*	$<0.05^{*}$	$<0.05^{*}$	>0.05	$<0.05^{*}$	$<0.05^{*}$
CTGAN	0.2346	0.1012	0.9136	0.8319	0.0653
DP-CTGAN	0.2553	0.0982	0.9880	0.8639	0.0654
*p-value*	$<0.05^{*}$	>0.05	$<0.05^{*}$	$<0.05^{*}$	>0.05
TVAE	0.2812	0.1190	0.6873	0.8709	0.0546
DP-TVAE	0.3010	0.1049	0.6823	0.8744	0.0653
*p-value*	$<0.05^{*}$	$<0.05^{*}$	$<0.05^{*}$	$<0.05^{*}$	>0.05
TabDif	0.3104	0.2430	0.5534	0.9589	0.2079
DP-TabDif	0.2402	0.1230	0.9104	0.8097	0.0625
*p-value*	$<0.05^{*}$	$<0.05^{*}$	$<0.05^{*}$	$<0.05^{*}$	$<0.05^{*}$

Bold values indicate statistically significant difference between the above model pairs.

STDG models trained without DP achieved significantly lower Hellinger distances across four out of five models (p<0.05), with GC (0.2124) and NPC (0.2193) preserving univariate distributions most effectively. The only model to improve with DP was DP-TabDif, reducing its distance from 0.3104 to 0.2402, while for the rest of the models the inclusion of DP had a negative impact to replicate univariate distributions. Conversely, TabDif (0.3104) and DP-TVAE (0.3010) showed the highest distances, highlighting a lower ability to preserve univariate distributions.

For PCD, NPC (PCD=0.0624) and DP-NPC (PCD=0.0627) models achieved the lowest values, with no significant difference between the two versions (p>0.05). DP-TVAE (PCD=0.1049) and DP-TabDif (PCD=0.1230) outperformed their non-DP counterparts in preserving correlations suggesting a positive impact of DP for correlations preservation. On the other hand, DP-GC (PCD=0.1562) showed a significant increase in PCD compared to GC (PCD=0.0668), reflecting a negative impact of DP on this model. The poorest correlation preservation was observed for TabDif (PCD=0.2430).

In terms of the $R^{2}$ of the DD-Plot, NPC ($R^{2}=0.9975$) and GC ($R^{2}=0.9301$) performed best without DP, while DP-CTGAN ($R^{2}=0.9880$) and DP-TabDif ($R^{2}=0.9104$) DP had a significantly positive impact (p<0.05), increasing the value. DP-NPC ($R^{2}=0.9114$) and DP-TVAE ($R^{2}=0.6823$) experienced reductions (p<0.05), suggesting a negative impact on dimensional fidelity when DP was added.

NPC (AUC−ROC=0.6568) and DP-NPC (AUC−ROC= 0.7418) were the only models to approach the ideal AUC-ROC of 0.5, indicating that the synthetic tabular data generated with these models was the least distinguishable from real data. All other models showed considerably higher scores, which increased significantly with DP, suggesting a negative impact of DP to generate synthetic samples indistinguishable from real samples.

Regarding the fidelity-utility tradeoff, statistically significant (p<0.05) and higher mean G values were observed for the NPC and GC models when adding DP, indicating a negative impact of DP for maintaining a tradeoff in the generated samples. In contrast, statistically significantly lower mean G values were obtained with TabDif when adding DP, indicating a positive impact. For CTGAN and TVAE, the addition of DP did not result in significant changes in the G metric when adding DP. Among all models, the lower G values were achieved by NPC (G=0.0211) and DP-NPC (G=0.0498), highlighting their capacity to balance fidelity and utility effectively.

#### 3.1.2 Utility

The classification and regression metrics differences between TRTR and TSTR for the Acute Myeolid Leukemia dataset are summarised in Table 4, showing the mean differences across the 10 evaluation folds. Figures S7, S9 of the Supplementary Material provides a visual representation of the variability and significance of these metrics across the STDG models. In most cases, statistically significant (p<0.05) and higher mean differences were observed for all classification and regression metrics when adding DP, indicating that DP negatively impacts the utility of the generated synthetic tabular data. However, the NPC model consistently exhibited the smallest differences, ranging from 0.0183 to 0.0385. Additionally, all models achieved metric differences below 0.3, suggesting that the utility of the synthetic data remains preserved to a reasonable extent across all STDG models.

**Table 4:** Utility results summary for the acute myeloid leukemia dataset.

Model	Classification metrics				Regression metrics			
	ACC dif.	PREC dif.	REC dif.	F1 dif.	MAE dif.	MSE dif.	RMSE dif.	$R^{2}$ dif.
NPC	0.0385	0.0316	0.0385	0.0356	0.0081	0.0042	0.0112	0.0183
DP-NPC	0.0589	0.0564	0.0589	0.0586	0.1146	0.1043	0.1795	0.0218
*p-value*	$<0.05^{*}$	$<0.05^{*}$	$<0.05^{*}$	$<0.05^{*}$	$<0.05^{*}$	$<0.05^{*}$	$<0.05^{*}$	>0.05
GC	0.0866	0.0456	0.0866	0.0730	0.0440	0.0193	0.0516	0.0316
DP-GC	0.2970	0.1179	0.2970	0.2524	0.0822	0.0465	0.1011	0.0773
*p-value*	$<0.05^{*}$	$<0.05^{*}$	$<0.05^{*}$	$<0.05^{*}$	$<0.05^{*}$	$<0.05^{*}$	$<0.05^{*}$	$<0.05^{*}$
CTGAN	0.1286	0.1383	0.1286	0.1347	0.0437	0.0208	0.0505	0.0777
DP-CTGAN	0.1424	0.1249	0.1424	0.1379	0.0347	0.0184	0.0454	0.0787
*p-value*	$<0.05^{*}$	$<0.05^{*}$	$<0.05^{*}$	>0.05	$<0.05^{*}$	$<0.05^{*}$	$<0.05^{*}$	>0.05
TVAE	0.0559	0.0558	0.0559	0.0552	0.0803	0.0793	0.1394	0.0548
DP-TVAE	0.0629	0.0614	0.0629	0.0582	0.1222	0.1538	0.2132	0.0624
*p-value*	$<0.05^{*}$	$<0.05^{*}$	$<0.05^{*}$	>0.05	$<0.05^{*}$	$<0.05^{*}$	$<0.05^{*}$	$<0.05^{*}$
TabDif	0.0501	0.0431	0.0501	0.0528	0.5419	0.3943	0.8258	0.0439
DP-TabDif	0.1219	0.1111	0.1219	0.1241	0.0417	0.0236	0.0527	0.0776
*p-value*	$<0.05^{*}$	$<0.05^{*}$	$<0.05^{*}$	$<0.05^{*}$	$<0.05^{*}$	$<0.05^{*}$	$<0.05^{*}$	$<0.05^{*}$

Bold values indicate statistically significant difference between the above model pairs.

#### 3.1.3 Privacy

The privacy risk measures for the Acute Myeloid Leukemia dataset, summarised in Table 5, demonstrate the significant impact of adding DP to decrease the privacy risks in certain models. In the table the mean differences across the 10 evaluation folds are shown and Figure S13 of the Supplementary Material provides a visual representation of the variability and significance of these measures across the STDG models.

**Table 5:** Privacy risk measures summary for the acute myeloid leukemia dataset.

Model	Linkability	Univariate singlingout	Multivariate singlingout	Membership inference	Attribute inference
NPC	0.1042	0.0006	0.1975	0.3060	0.3976
DP-NPC	0.0245	0.0006	0.1154	0.0651	0.2739
*p-value*	$<0.05^{*}$	>0.05	$<0.05^{*}$	$<0.05^{*}$	$<0.05^{*}$
GC	0.0009	0.0082	0.1758	0.0	0.0356
DP-GC	0.0003	0.0076	0.1265	0.0	0.0177
*p-value*	>0.05	>0.05	$<0.05^{*}$	>0.05	>0.05
CTGAN	0.0009	0.0042	0.1253	0.0003	0.0282
DP-CTGAN	0.0003	0.0129	0.1264	0.0	0.0216
*p-value*	>0.05	$<0.05^{*}$	>0.05	>0.05	>0.05
TVAE	0.0006	0.0060	0.0833	0.0016	0.0394
DP-TVAE	0.0006	0.0085	0.0944	0.0003	0.0386
*p-value*	>0.05	>0.05	>0.05	>0.05	>0.05
TabDif	0.0019	0.0	0.0	0.0032	0.0641
DP-TabDif	0.0032	0.0	0.0475	0.0009	0.0439
*p-value*	>0.05	>0.05	>0.05	$<0.05^{*}$	$<0.05^{*}$

Bold values indicate statistically significant difference between the above model pairs.

For linkability attack, DP significantly reduced risks in NPC from 0.1042 (NPC) to 0.0245 (DP-NPC), while no significant reductions were observed for GC, CTGAN, TVAE and TabDif in their DP counterpart. However, all models achieved linkability risks below 0.1, indicating minimal risk of linking synthetic samples to real ones for all models.

In univariate singlingout, risks were low across all models (<0.02), with a significant increase only for CTGAN, from 0.0042 (CTGAN) to 0.0129 (DP-CTGAN), when adding DP. No other models showed significant differences in their corresponding DP counterparts. For multivariate singlingout, DP significantly reduced risks for NPC from 0.1975 (NPC) to 0.1154 (DP-NPC). For the other models, risks remained low (<0.2) without significant difference in their DP counterpart, indicating a generally low likelihood of identifying samples with specific attribute combinations.

In the membership inference attack, NPC showed the highest risk (0.3060), which dropped significantly with DP-NPC (0.0651). All other models maintained very low risks (ranging from 0.0 to 0.0032), with a significant risk decrease only for TabDif, from 0.0032 (TabDif) to 0.0009 (DP-TabDif). Therefore, all models except NPC were able to generate synthetic samples that cannot be linked to an existing sample in the real data.

Finally, for attribute inference attacks, DP significantly reduced risks in NPC, from 0.3976 (NPC) to 0.2739 (DP-NPC), and TabDif, from 0.0641 (TabDif) to 0.0439 (DP-TabDif). The rest of the models showed consistently low risks (<0.08), indicating minimal vulnerability to attribute linking.

### 3.2 Brain stroke dataset

#### 3.2.1 Fidelity and tradeoff

The fidelity and tradeoff metrics for the Brain Stroke dataset are summarised in Table 6, showing the mean values across the 10 evaluation folds. Figure S18 of the Supplementary Material provides a visual representation of the variability and significance of these metrics across the STDG models.

**Table 6:** Fidelity and tradeoff results summary for brain stroke dataset.

Model	Hellinger distance	PCD	$R^{2}$ of DD-plot	AUC-ROC	Tradeoff (G)
NPC	0.1593	0.0119	0.9989	0.5332	0.0224
DP-NPC	0.2282	0.0400	0.9984	0.5752	0.0429
*p-value*	$<0.05^{*}$	$<0.05^{*}$	>0.05	$<0.05^{*}$	$<0.05^{*}$
GC	0.1614	0.1308	0.9599	0.6793	0.0695
DP-GC	0.2835	0.1593	0.9608	0.9175	0.1318
*p-value*	$<0.05^{*}$	$<0.05^{*}$	>0.05	$<0.05^{*}$	$<0.05^{*}$
CTGAN	0.1365	0.0613	0.9958	0.6195	0.0421
DP-CTGAN	0.1465	0.0726	0.9916	0.6384	0.0469
*p-value*	$<0.05^{*}$	$<0.05^{*}$	$<0.05^{*}$	$<0.05^{*}$	>0.05
TVAE	0.1733	0.1064	0.9474	0.6532	0.0534
DP-TVAE	0.2408	0.1375	0.8670	0.7560	0.0677
*p-value*	$<0.05^{*}$	$<0.05^{*}$	$<0.05^{*}$	$<0.05^{*}$	$<0.05^{*}$
TabDif	0.2203	0.1597	0.6398	0.9217	0.1345
DP-TabDif	0.3127	0.2056	0.4876	0.9544	0.2538
*p-value*	$<0.05^{*}$	$<0.05^{*}$	$<0.05^{*}$	$<0.05^{*}$	$<0.05^{*}$

Bold values indicate statistically significant difference between the above model pairs.

STDG models trained without DP achieved significantly lower Hellinger distances for the five models (p<0.05), with CTGAN (0.1365) and NPC (0.1593) preserving univariate distributions most effectively. Conversely, DP-TabDif (0.3127) and DP-GC (0.2835) showed the highest mean Hellinger distances, highlighting a lower ability to preserve univariate distributions. When adding DP to the models, significantly higher Hellinger distances were obtained, suggesting the negative impact of DP for preserving univariate distributions.

For PCD, NPC (PCD=0.0119) and DP-NPC (PCD=0.04) models achieved the lowest values, with a significant difference between the two versions (p<0.05). In general, all models obtained significantly higher PCD values (p<0.05) when adding DP, reflecting a negative impact of DP when preserving pairwise correlations. The poorest correlation preservation was observed for DP-TabDif (PCD=0.2056).

In terms of the $R^{2}$ of the DD-Plot, NPC ($R^{2}=0.9989$), CTGAN ($R^{2}=0.9958$) and GC ($R^{2}=0.9599$) performed best without DP, while DP-NPC ($R^{2}=0.9984$) and DP-GC ($R^{2}=0.9608$) did not have any significant impact under DP constraints (p>0.05). For DP-CTGAN ($R^{2}=0.9916$), DP-TVAE ($R^{2}=0.8670$) and DP-TabDif ($R^{2}=0.4876$), the addition of DP impacted negatively, suggesting a loss of dimensional fidelity when DP was added.

NPC (AUC−ROC=0.5333), DP-NPC (AUC−ROC= 0.5752), CTGAN (AUC−ROC=0.6195) and DP-CTGAN ((AUC−ROC=0.6385) were the only models approaching the ideal AUC-ROC of 0.5, indicating that the synthetic tabular data generated by these models was the least distinguishable from real data. All other models showed considerably higher scores, which increased significantly with DP, suggesting a negative impact of DP on the distinguishability of synthetic tabular data.

Regarding the fidelity-utility tradeoff, statistically significantly (p<0.05) and higher mean G values were observed for the NPC, GC, TVAE and TabDif models when adding DP, indicating a negative impact of DP for fidelity-utility tradeoff. For CTGAN, the addition of DP did not result in significant changes in the G metric when adding DP. Among all models, the lower G values were achieved by NPC (G=0.0224), DP-NPC (G=0.0429), CTGAN (G=0.0421), and DP-CTGAN (G=0.0469) highlighting their capacity to balance fidelity and utility effectively.

#### 3.2.2 Utility

The classification and regression metrics differences between TRTR and TSTR for the Brain Stroke dataset are summarised in Table 7, showing the mean values for the 10 folds and Figures S20, S22 of the Supplementary Material provides a visual representation of the variability and significance of these metrics across the STDG models. In all STDG models except CTGAN and DP-CTGAN, statistically significant (p<0.05) and higher mean differences were observed for all classification and regression metrics when adding DP, indicating that DP negatively impacts the utility of the generated synthetic tabular data in most models. However, the NPC model consistently exhibited the smallest differences, ranging from 0.0088 to 0.0513. Additionally, all models except TabDif and DP-TabDif achieved metric differences below 0.3, suggesting that the utility of the synthetic data remains preserved to a reasonable extent most STDG models.

**Table 7:** Utility results summary for the brain stroke dataset.

Model	Classification metrics				Regression metrics			
	ACC dif.	PREC dif.	REC dif.	F1 dif.	MAE dif.	MSE dif.	RMSE dif.	$R^{2}$ dif.
NPC	0.0513	0.0424	0.0513	0.0513	0.0101	0.0031	0.0088	0.0492
DP-NPC	0.0702	0.0588	0.0702	0.0732	0.0503	0.0200	0.0584	0.1385
*p-value*	$<0.05^{*}$	$<0.05^{*}$	$<0.05^{*}$	$<0.05^{*}$	$<0.05^{*}$	$<0.05^{*}$	$<0.05^{*}$	$<0.05^{*}$
GC	0.1104	0.0926	0.1105	0.0531	0.0471	0.0157	0.0480	0.1472
DP-GC	0.3060	0.1015	0.3060	0.0732	0.0712	0.0307	0.0787	0.2329
*p-value*	$<0.05^{*}$	$<0.05^{*}$	$<0.05^{*}$	$<0.05^{*}$	$<0.05^{*}$	$<0.05^{*}$	$<0.05^{*}$	$<0.05^{*}$
CTGAN	0.0890	0.0688	0.0890	0.0866	0.0278	0.0089	0.0310	0.951
DP-CTGAN	0.0933	0.0672	0.0933	0.0910	0.0266	0.0089	0.0301	0.0869
*p-value*	>0.05	>0.05	>0.05	>0.05	>0.05	>0.05	>0.05	$<0.05^{*}$
TVAE	0.0870	0.0649	0.0870	0.0842	0.0385	0.1644	0.0490	0.1373
DP-TVAE	0.1079	0.7389	0.1079	0.1070	0.0521	0.0224	0.0667	0.1694
*p-value*	$<0.05^{*}$	$<0.05^{*}$	$<0.05^{*}$	$<0.05^{*}$	$<0.05^{*}$	$<0.05^{*}$	$<0.05^{*}$	$<0.05^{*}$
TabDif	0.1142	0.0662	0.1142	0.0997	0.3808	0.3941	0.5909	0.1132
DP-TabDif	0.1828	0.2292	0.1828	0.2349	0.6902	0.5744	0.6154	0.1862
*p-value*	$<0.05^{*}$	$<0.05^{*}$	$<0.05^{*}$	$<0.05^{*}$	$<0.05^{*}$	$>0.05^{*}$	$>0.05^{*}$	$<0.05^{*}$

Bold values indicate statistically significant difference between the above model pairs.

#### 3.2.3 Privacy

The privacy risk measures for the Brain Stroke dataset, summarised in Table 8, showed that adding DP to the STDG models had no significant impact on privacy risks for most STDG models and privacy attacks. In the table, the mean differences across the 10 evaluation folds are shown and Figure S26 of the Supplementary Material provides a visual representation of the variability and significance of these measures across the STDG models.

**Table 8:** Privacy risk measures summary for the brain stroke dataset.

Model	Linkability	Univariate singlingout	Multivariate singlingout	Membership inference	Attribute inference
NPC	0.0007	0.0	0.1129	0.5642	0.2313
DP-NPC	0.0007	0.0	0.1138	0.3042	0.1961
*p-value*	>0.05	>0.05	>0.05	$<0.05^{*}$	$<0.05^{*}$
GC	0.0	0.0598	0.0701	0.0530	0.0260
DP-GC	0.0	0.0550	0.0611	0.0234	0.0172
*p-value*	>0.05	>0.05	>0.05	>0.05	$<0.05^{*}$
CTGAN	0.0	0.0212	0.0862	0.0472	0.0259
DP-CTGAN	0.0	0.0589	0.0727	0.0074	0.0251
*p-value*	>0.05	>0.05	>0.05	>0.05	>0.05
TVAE	0.0	0.0712	0.0904	0.0177	0.0359
DP-TVAE	0.0001	0.0614	0.0140	0.0398	0.0395
*p-value*	>0.05	>0.05	$<0.05^{*}$	>0.05	>0.05
TabDif	0.0	0.0	0.0080	0.0131	0.0267
DP-TabDif	0.0032	0.0	0.0	0.0109	0.0387
*p-value*	$<0.05^{*}$	>0.05	$<0.05^{*}$	>0.05	>0.05

Bold values indicate statistically significant difference between the above model pairs.

An exception was observed in the linkability attack, where DP significantly increased the risk for TabDif, from 0.0 (TabDif) to 0.0032 (DP-TabDif). For all other STDG models, no significant changes in linkability risks were observed between non-DP and DP versions. Additionally, all models achieved linkability risks below 0.0008, highlighting a very minimal risk of linking synthetic samples to real ones for all models.

In univariate singlingout attacks, no significant differences were found between non-DP and DP model versions, with risks remaining low (<0.062). In contrast, multivariate singlingout attack risks were significantly reduced when adding DP for TVAE, from 0.0904 (TVAE) to 0.0140 (DP-TVAE), and for TabDif, from 0.008 (TabDif) to 0.0 (DP-TabDif). For the rest of the models, the multivariate singlingout attack risks were slightly higher, ranging from 0.1138 (DP-NPC) to 0.0611 (DP-GC). These results suggest a generally low likelihood of identifying samples with specific attribute combinations for all STDG models.

Membership inference attack risks, were highest for NPC (0.5642), which was significantly reduced for DP-NPC (0.3042). All other models exhibited lower risks, ranging from 0.0109 (DP-TabDif) to 0.0530 (GC), with no significant changes between non-DP and DP model versions. These results demonstrate that, except for NPC, all models effectively generated synthetic samples that cannot be linked to existing samples in the real data.

Finally, for attribute inference attacks, DP significantly reduced risks in NPC, from 0.2313 (NPC) to 0.1961 (DP-NPC), and GC, from 0.0260 (GC) to 0.0172 (DP-GC). The remaining models maintained consistently low risks (<0.04), indicating minimal vulnerability to attribute linking.

### 3.3 Cardiovascular disease dataset

#### 3.3.1 Fidelity and tradeoff

The fidelity and tradeoff metrics for the Cardiovascular Disease dataset are summarised in Table 9, showing the mean values across the 10 evaluation folds. Figure S31 of the Supplementary Material provides a visual representation of the variability and significance of these metrics across the STDG models.

**Table 9:** Fidelity and tradeoff results summary for the cardiovascular disease dataset.

Model	Hellinger distance	PCD	$R^{2}$ of DD-plot	AUC-ROC	Tradeoff (G)
NPC	0.2539	0.0115	0.9911	0.9462	0.0168
DP-NPC	0.2543	0.0131	0.9949	0.9474	0.0261
*p-value*	$<0.05^{*}$	$<0.05^{*}$	>0.05	$<0.05^{*}$	$<0.05^{*}$
GC	0.2779	0.0412	0.9962	0.9234	0.0355
DP-GC	0.3974	0.1149	0.9953	0.9560	0.1138
*p-value*	$<0.05^{*}$	$<0.05^{*}$	>0.05	$<0.05^{*}$	$<0.05^{*}$
CTGAN	0.2903	0.0791	0.8960	0.7817	0.0427
DP-CTGAN	0.2905	0.0765	0.8210	0.7746	0.0555
*p-value*	>0.05	$<0.05^{*}$	$<0.05^{*}$	$<0.05^{*}$	$<0.05^{*}$
TVAE	0.3200	0.1572	0.0	0.8170	0.0800
DP-TVAE	0.3164	0.2062	0.0	0.8815	0.1221
*p-value*	>0.05	$<0.05^{*}$	>0.05	$<0.05^{*}$	$<0.05^{*}$
TabDif	0.3592	0.1585	0.3701	0.9643	0.4898
DP-TabDif	0.4084	0.2073	0.3524	0.9789	0.0335
*p-value*	$<0.05^{*}$	$<0.05^{*}$	$<0.05^{*}$	$<0.05^{*}$	$<0.05^{*}$

Bold values indicate statistically significant difference between the above model pairs.

STDG models trained without DP achieved significantly lower Hellinger distances for all STDG models (p<0.05), indicating a negative impact of DP on preserving univariate distributions. However, CTGAN and TVAE showed no significant differences in Hellinger distance when DP was added to the model. NPC (0.2539), DP-NPC (0.2543) and GC (0.2779) were the models that best preserved univariate distributions, achieving the lowest distance values. In contrast, DP-TabDif (0.4084), DP-GC (0.3974) and TabDif (0.3592) demonstrated the poorest performance in preserving univariate distributions, with the highest distance values.

Regarding the PCD, NPC (PCD=0.0115) and DP-NPC (PCD=0.0131) achieved the lowest values, with a significant difference between the two versions (p<0.05). In general, all models obtained significantly higher PCD values (p<0.05) when adding DP, reflecting a negative impact of DP when preserving pairwise correlations. However, DP-CTGAN (PCD=0.0765) showed significantly lower PCD value than CTGAN (PCD=0.0791). The poorest correlation preservation were observed for DP-TabDif (PCD=0.2073), TabDif (PCD=0.1585) and TVAE (PCD=0.1572).

In terms of the $R^{2}$ of the DD-Plot, GC ($R^{2}=0.9962$) and NPC ($R^{2}=0.9911$) performed best without DP, while DP-GC ($R^{2}=0.9949$) and DP-NPC ($R^{2}=0.9949$) did not have any significant impact under DP constraints (p>0.05). For DP-CTGAN ($R^{2}=0.8210$) and DP-TabDif ($R^{2}=0.3524$), the addition of DP impacted negatively, suggesting a loss of dimensional fidelity when DP was added. The worst dimensional fidelity was obtained with TVAE and DP-TVAE ($R^{2}=0$ for both models), with no significant difference between the DP and non-DP versions.

Despite being a bit far, CTGAN (AUC−ROC=0.7817), DP-CTGAN (AUC−ROC=0.7746) were the only models approaching the ideal AUC-ROC of 0.5, indicating that the synthetic tabular data generated by these models was the least distinguishable from real data. All other models showed considerably higher scores, which increased significantly with DP, suggesting a negative impact of DP on the distinguishability of synthetic tabular data.

Regarding the fidelity-utility tradeoff, statistically significantly (p<0.05) and higher mean G values were observed for the NPC, GC, TVAE and CTGAN models when adding DP, indicating a negative impact of DP for fidelity-utility tradeoff. For TabDif, the addition of DP resulted in a significant reduction of the G metric when adding DP. Among all models, the lower G values were achieved by NPC (G=0.0168) and DP-NPC (G=0.0261), while the higher G values were achieved by TabDif (G=0.4898) and DP-TVAE (G=0.1221).

#### 3.3.2 Utility

The classification and regression metric differences between TRTR and TSTR for the Cardiovascular Disease dataset are summarised in Table 10, showing the mean values for the 10 folds and Figures S33, S35 of the Supplementary Material provides a visual representation of the variability and significance of these metrics across the STDG models. Statistically significant (p<0.05) mean differences were observed for all classification and regression metrics in GC and TabDif, with higher differences for DP-GC and lower differences for DP-TabDif. These results indicate that DP negatively impacted the utility of synthetic tabular data generated by DP-GC, while it had a positive impact for DP-TabDif. For the remaining models (NPC, CTGAN, and TVAE) no statistically significant (p>0.05) mean differences were observed for most classification and regression metrics, suggesting that DP had no significant impact on the utility of the generated synthetic tabular data. NPC consistently exhibited the smallest differences, ranging from 0.001 to 0.0582. Additionally, all models except TabDif achieved metric differences below 0.25, indicating that the utility of synthetic data remains well-preserved for most STDG models.

**Table 10:** Utility results summary for the cardiovascular disease dataset.

Model	Classification metrics				Regression metrics			
	ACC dif.	PREC dif.	REC dif.	F1 dif.	MAE dif.	MSE dif.	RMSE dif.	$R^{2}$ dif.
NPC	0.0532	0.0271	0.0532	0.0582	0.0061	0.0010	0.0062	0.0196
DP-NPC	0.0539	0.0309	0.0539	0.0588	0.0547	0.0246	0.0812	0.0175
*p-value*	>0.05	$<0.05^{*}$	>0.05	>0.05	$<0.05^{*}$	$<0.05^{*}$	$<0.05^{*}$	>0.05
GC	0.0864	0.0386	0.0846	0.0785	0.0417	0.0158	0.0726	0.0266
DP-GC	0.3745	0.0982	0.3744	0.3608	0.0742	0.0341	0.1138	0.0499
*p-value*	$<0.05^{*}$	$<0.05^{*}$	$<0.05^{*}$	$<0.05^{*}$	$<0.05^{*}$	$<0.05^{*}$	$<0.05^{*}$	$<0.05^{*}$
CTGAN	0.0657	0.0356	0.0657	0.0626	0.0372	0.0787	0.1496	0.0336
DP-CTGAN	0.0693	0.0336	0.0693	0.0692	0.0369	0.2184	0.2238	0.0323
*p-value*	>0.05	>0.05	>0.05	0.05	>0.05	$<0.05^{*}$	$<0.05^{*}$	>0.05
TVAE	0.1159	0.0406	0.1159	0.0949	0.0597	0.1509	0.2150	0.0281
DP-TVAE	0.1137	0.0501	0.1137	0.0993	0.1090	0.5197	0.4631	0.0268
*p-value*	>0.05	$<0.05^{*}$	>0.05	>0.05	$<0.05^{*}$	$<0.05^{*}$	$<0.05^{*}$	>0.05
TabDif	0.0897	0.0511	0.0897	0.0905	0.8206	0.5662	0.5881	0.0380
DP-TabDif	0.1253	0.1009	0.1253	0.1326	0.4057	0.0103	0.0711	0.0415
*p-value*	$<0.05^{*}$	$<0.05^{*}$	$<0.05^{*}$	$<0.05^{*}$	$<0.05^{*}$	$<0.05^{*}$	$<0.05^{*}$	$<0.05^{*}$

Bold values indicate statistically significant difference between the above model pairs.

#### 3.3.3 Privacy

The privacy risk measures for the Cardiovascular Disease dataset, summarised in Table 11, showed that adding DP to the STDG models generally had no significant impact on privacy risks for most STDG models and privacy attacks. In the table, the mean differences across the 10 evaluation folds are shown and Figure S39 of the Supplementary Material provides a visual representation of the variability and significance of these measures across the STDG models.

**Table 11:** Privacy risk measures summary for the cardiovascular disease dataset.

Model	Linkability	Univariate singlingout	Multivariate singlingout	Membership inference	Attribute inference
NPC	0.0012	0.0	0.0554	0.3938	0.1752
DP-NPC	0.0	0.0	0.0761	0.0823	0.0702
*p-value*	$<0.05^{*}$	>0.05	>0.05	$<0.05^{*}$	$<0.05^{*}$
GC	0.0	0.1722	0.0758	0.0304	0.0279
DP-GC	0.0	0.1217	0.0671	0.0002	0.0132
*p-value*	>0.05	>0.05	>0.05	$<0.05^{*}$	$<0.05^{*}$
CTGAN	0.0	0.1886	0.0770	0.0413	0.0362
DP-CTGAN	0.0	0.1449	0.1147	0.0750	0.0293
*p-value*	>0.05	>0.05	$<0.05^{*}$	>0.05	>0.05
TVAE	0.0	0.3176	0.0451	0.0059	0.0311
DP-TVAE	0.0	0.3567	0.0276	0.0122	0.0330
*p-value*	>0.05	>0.05	$<0.05^{*}$	>0.05	>0.05
TabDif	0.0	0.1810	0.0	0.0024	0.0317
DP-TabDif	0.0017	0.1787	0.0	0.0039	0.0408
*p-value*	$<0.05^{*}$	>0.05	>0.05	>0.05	>0.05

Bold values indicate statistically significant difference between the above model pairs.

In the linkability attack, DP significantly reduced the risk for NPC, from 0.0012 (NPC) to 0.0 (DP-NPC), while it significantly increased the risk for TabDif, from 0.0 (TabDif) to 0.0017 (DP-TabDif). For all other STDG models, no significant changes were observed between non-DP and DP versions, with all models achieving linkability risks of 0.0, highlighting negligible risk of linking synthetic samples to real ones.

For univariate singlingout attacks, no significant differences were found between non-DP and DP model versions, with risks remaining low (<0.2) across all STDG models except TVAE (0.3176) and DP-TVAE (0.3567). In contrast, multivariate singlingout attack risks increased significantly for CTGAN when adding DP, rising from 0.0770 (CTGAN) to 0.1147 (DP-CTGAN), while risks decreased significantly for TVAE, from 0.0451 (TVAE) to 0.0276 (DP-TVAE). For the rest of the models, multivariate singlingout risks did not significantly change when adding DP, ranging from 0.0 (TabDif and DP-TabDif) to 0.0761 (DP-NPC), indicating a low likelihood of identifying samples with specific attribute combinations for all STDG models.

Membership inference attack risk was highest for NPC (0.3938), but was significantly reduced for DP-NPC (0.0832). Similarly, DP significantly reduced risks for GC, from 0.0304 (GC) to 0.0002 (DP-GC). For all other models, risks remained low, ranging from 0.0024 (DP-TabDif) to 0.0750 (DP-CTGAN), with no significant changes between non-DP and DP model versions. These results demonstrate that, apart from NPC, all models effectively generated synthetic samples that could not be linked to existing samples in the real data.

Finally, for attribute inference attacks, DP significantly reduced risks in NPC, from 0.1752 (NPC) to 0.0702 (DP-NPC), and in GC, from 0.0279 (GC) to 0.0132 (DP-GC). The remaining models maintained consistently low risks (<0.05), indicating minimal vulnerability to attribute linking.

### 3.4 Results summary

The obtained results across all evaluation categories and datasets are summarised in Table 12 summarised, highlighting the best and worst STDG models and the impact of DP in each evaluation category for each dataset. The impact of DP is classified as positive if it significantly improves the majority of metrics or privacy risk measures within a category, negative if it significantly worsens them, and neutral if no significant changes are observed (p>0.05).

**Table 12:** Summary of best STDG models and DP impact across datasets for each dimension and overall performance.

Dataset	Category	Best model	Worst model	DP impact
Acute myeloid leukemia dataset (26)	Fidelity	NPC	TabDif	∙ Negative: DP-NPC, DP-GC, DP-CTGAN and DP-TVAE ∙ Positive: DP-TabDif
	Utility	NPC	DP-CTGAN	∙ Negative: all models
	Tradeoff	NPC	TabDif	∙ Negative: DP-NPC and DP-GC ∙ Positive: DP-TabDif ∙ Neutral: DP-CTGAN and DP-TVAE
	Privacy	DP-TabDif	NPC	∙ Negative: DP-CTGAN ∙ Positive: DP-NPC and DP-TabDif ∙ Neutral: DP-GC and DP-TVAE
Brain stroke dataset (27)	Fidelity	NPC	DP-TabDif	∙ Negative: all models
	Utility	NPC	DP-TabDif	∙ Negative: DP-NPC, DP-GC, DP-TVAE and DP-TabDif ∙ Neutral: DP-CTGAN
	Tradeoff	NPC	DP-TabDif	∙ Negative: DP-NPC, DP-GC, DP-TVAE and DP-TabDif ∙ Neutral: DP-CTGAN
	Privacy	TabDif	NPC	∙ Positive: DP-NPC ∙ Neutral: DP-GC, DP-CTGAN, DP-TVAE and DP-TabDif
Cardiovascular disease dataset (28)	Fidelity	NPC	DP-TabDif	∙ Negative: DP-NPC, DP-GC, DP-TabDif ∙ Positive: DP-CTGAN ∙ Neutral: DP-TVAE
	Utility	NPC	TabDif	∙ Negative: DP-GC ∙ Positive: DP-TabDif ∙ Neutral: DP-NPC, DP-CTGAN, DP-TVAE
	Tradeoff	NPC	TabDif	∙ Negative: DP-NPC, DP-GC, DP-TVAE and DP-CTGAN ∙ Positive: DP-TabDif
	Privacy	DP-GC	NPC	∙ Positive: DP-NPC ∙ Neutral: DP-GC, DP-CTGAN, DP-TVAE and DP-TabDif

#### 3.4.1 Acute myeolid leukemia dataset

For the Acute Myeolid Leukemia dataset, NPC was the most balanced model, achieving high fidelity, maintaining utility, and demonstrating the best tradeoff between fidelity and utility, though it exhibited the highest privacy risks. The addition of DP negatively impacted the utility of all models and the fidelity of most models, except for DP-TabDif, which showed improvements in both fidelity and tradeoff. TabDif had the poorest fidelity and tradeoff results, making it the worst model overall. In terms of privacy, DP-TabDif achieved the lowest attack risks that were reduced when adding DP, as did DP-NPC. In contrast, DP-CTGAN performed worst in preserving the utility of synthetic tabular data and was the only STDG model where DP negatively impacted privacy risks. These findings highlight the improvement of TabDif when adding DP (DP-TabDif) in synthetic tabular data quality while reducing privacy risks and the consistent performance of NPC across all categories except privacy.

#### 3.4.2 Brain stroke dataset

For the Brain Stroke dataset, NPC was the most balanced model, achieving high fidelity, maintaining utility, and demonstrating the best tradeoff between fidelity and utility, although it exhibited the highest privacy risks. The addition of DP negatively impacted the fidelity of all models, as well as the utility and tradeoff of most models. DP-TabDif demonstrated the poorest performance across fidelity, utility and tradeoff, making it the weakest model overall. Regarding privacy, TabDif achieved the lowest attack risks and for DP-NPC, the incorporation of DP significantly reduced privacy risks. These findings underscore the consistent performance of NPC across all categories except privacy and highlight the positive effect of adding DP to this model (DP-NPC) in reducing privacy risks. For the remaining models and most categories, the addition of DP generally had no impact or a negative impact.

#### 3.4.3 Cardiovascular disease dataset

For the Cardiovascular Disease dataset, NPC emerged as the most balanced model, achieving high fidelity, maintaining utility, and demonstrating the best tradeoff between fidelity and utility, unless it obtained the highest privacy risks. TabDif and DP-TabDif performed poorest across fidelity, utility and tradeoff, making them the weakest models overall. Adding DP negatively impacted the fidelity of DP-NPC, DP-GC and DP-TabDif, while it positively impacted DP-CTGAN. Regarding utility, only DP-TabDif was improved when adding DP, while DP-GC was worsened. For the fidelity-utility tradeoff, DP had a negative impact across all STDG models except DP-TabDif, which benefited from a positive impact. In terms of privacy, DP-GC achieved the lowest attack risks, while DP-NPC had a positive impact on privacy risks when adding DP. These findings highlight the consistent performance of NPC across all categories except privacy and emphasize the positive effect of adding DP to this model (DP-NPC) in reducing privacy risks. For the remaining models and most categories, the impact of DP varied depending on the category.

## 4 Discussion

### 4.1 Main findings

By applying the proposed evaluation framework to synthetic tabular data generated by STDG models with and without privacy guarantees (specifically, DP) across three open-source medical datasets with varying characteristics, the results demonstrated that the proposed synthetic tabular data evaluation framework can effectively be used to assess and compare synthetic tabular data. The applied methodology, which simulated cross-validation and evaluated significant differences between model pairs (non-DP and DP versions), successfully validated the robustness of the framework. This approach enabled a detailed and holistic analysis of the impact of incorporating the DP mechanism into different STDG model typologies and the suitability of these models for medical datasets with diverse characteristics. Unlike previous frameworks proposed by Hernandez et al. (13), Lautrup et al. (14) and Livieris et al. (15), the presented evaluation framework provides a holistic evaluation of synthetic tabular data by consolidating a minimal yet robust set of metrics and privacy risk measures focused on fidelity, utility, and privacy, while avoiding redundancy. Additionally, it incorporates the metric introduced by Galloni et al. (21), which quantifies the tradeoff between fidelity and utility, offering deeper insights into the performance of the STDG model.

### 4.2 STDG models ranking

For the three datasets, NPC was the most balanced model, achieving high fidelity, maintaining utility, and demonstrating the best tradeoff between fidelity and utility. However, NPC exhibited the highest privacy risks for all datasets, being the worst model to preserve the privacy of real data. This finding suggests that NPC has been the most effective model for data augmentation but the worst for privacy preservation. On the other hand, TabDif and DP-TabDif were the worst models to preserve fidelity, and utility and demonstrate a tradeoff between fidelity and utility for the three datasets. However, DP-TabDif, TabDif and DP-GC exhibited the lowest privacy risks for the Acute Myeloid Leukemia dataset, and Cardiovascular Disease dataset respectively, suggesting that they have been the best STDG models for privacy preservation. DP-CTGAN was the worst model for utility preservation in the Acute Myeloid Leukemia dataset.

### 4.3 DP impact on STDG models

The incorporation of DP generally worsened the fidelity of the synthetic tabular data across most STDG models and dataset combinations. Specifically, fidelity metrics got worse for DP-NPC and DP-GC models across all three datasets, highlighting the negative impact of DP-induced noise on preserving statistical properties. However, there were exceptions: DP-TabDif improved fidelity for the Acute Myeloid Leukemia dataset, and DP-CTGAN for the Cardiovascular Disease dataset. For the Brain Stroke dataset, fidelity consistently was deteriorated across all DP-integrated models. Apart from that, for the Cardiovascular Disease dataset, the addition of DP to DP-TVAE neither improved nor worsened fidelity, demonstrating a neutral impact. These findings suggest that while DP introduces noise to the generated synthetic samples, this often reduces their fidelity.

Regarding the utility of synthetic tabular data, the integration of DP into the STDG models generally was also negatively affected across most STDG models and dataset combinations. For the Acute Myeloid dataset, utility consistently reduced across all models when DP was integrated. Similarly, for the Brain Stroke dataset, most models were not capable of preserving synthetic data utility when adding DP, except DP-CTGAN, which neither reduced nor improved utility. A unique exception was observed for the Cardiovascular Disease dataset, where utility deteriorated with DP-GC but improved for DP-TabDif, while the addition of DP had no significant impact on the utility of other models. These findings suggest that the noise introduced by DP frequently diminishes or, at best, does not affect the utility of synthetic tabular data.

The impact of adding DP to the STDG models on the fidelity-utility tradeoff aligned closely with its effects on fidelity and utility, generally worsening for most STDG models and dataset combinations. Consistent with fidelity results, the tradeoff deteriorated for synthetic tabular data generated by DP-NPC and DP-GC models across all three datasets. However, DP-TabDif demonstrated an improvement in the tradeoff for both the Acute Myeloid Leukemia dataset and the Cardiovascular Disease dataset, highlighting its adaptability under certain conditions. These findings suggest that while DP introduces noise to enhance privacy, this often comes at the cost of fidelity and utility, underscoring the challenge of balancing privacy and fidelity-utility tradeoff in synthetic tabular data generation.

Contrary to expectations, adding DP to the STDG models neither significantly reduced nor increased privacy risks for most STDG models and dataset combinations. The only model that significantly reduced privacy risks across all datasets when incorporating DP was DP-NPC. For the other models, the addition of DP generally had no noticeable impact on privacy risks. An exception was observed for the Acute Leukemia dataset, where DP-CTGAN increased privacy risks, whereas DP-TabDif reduced them. These findings suggest that incorporating DP into selected STDG models did not reduce privacy risks as expected; similar levels of privacy risk were observed for both the non-DP and DP counterparts across most model and dataset combinations.

In general, fidelity was the category most affected by the addition of DP to the STDG models, while privacy was the least affected. This suggests that, based on the performed evaluations and analysis, unless DP introduces noise in the STDG models to reduce privacy risks in synthetic tabular data, the fidelity and utility of synthetic data are negatively affected, with no significant reduction in privacy risks observed. Therefore, further research and evaluation are needed to determine whether incorporating DP into STDG models for generating synthetic tabular data is justified, as it compromised the fidelity and utility of the generated samples without significantly mitigating privacy risks.

### 4.4 Limitations and future work

While this paper presents a valuable evaluation framework for synthetic tabular data in three main categories (fidelity, utility and privacy) and the fidelity-utility tradeoff, applied to three open-source medical datasets to asses STDG models with and without privacy guarantees (specifically, DP), there are several limitations and future directions that warrant further refinement and exploration of the framework.

One key limitation of this framework is the lack of additional evaluation categories beyond fidelity, utility and privacy. While these three core categories are widely used to evaluate the quality and risk of synthetic tabular data, other aspects more related to the efficiency of STDG models, such as, training time, resource consumption, generalization capability and model interpretability, could also be relevant. The incorporation of such type of metrics and measures can provide a more comprehensive analysis of the computational costs associated with the training of different STDG models and parameter configurations. Similarly, exploring interpretability or generalization of used STDG models could provide further insights for generative model selection for different use cases. Future research should consider extending the proposed evaluation framework to include these complementary evaluation categories, and to perform an exhaustive comparison focused on time efficiency, resource consumption and evaluation outcomes between this framework and other available synthetic tabular data evaluation frameworks. The evaluation framework should also include more tradeoff metrics, for example, fidelity-privacy tradeoff, that can provide more insights about the capability of the model to generate synthetic tabular with low privacy risk without impacting too much the fidelity.

The applied methodology of generating and evaluating 10 folds and averaging the results to validate the efficacy of the framework was particularly time-consuming in many cases, highlighting the need for optimizing or paralleling the computation of the evaluation metrics and privacy risk measures. Additionally, to reduce computational costs, the train test split was randomly performed only once at the beginning of the evaluation flow, thus avoiding the need to train each STDG model on multiple training folds. However, the representativeness and consistency of the train and test folds were not explicitly verified, and using a single train fold does not account for potential variability introduced by different data partitions. Therefore, future work could explore the use of multiple train and test splits, along with the validation of their consistency by comparing descriptive statistics and distributional properties.

Another limitation of this methodology lies in the parameter configuration for the used STDG models. With the objective to ensure methodological consistency across experiments and to maintain computational feasibility, all STDG models were trained using default parameters, which may not represent their optimal performance. While this approach allows for fair comparisons between non-DP and DP counterparts of each model, it may not reflect the optimal performance of individual models. Thus, future research should follow the work developed by Du et al. (39), which highlights the benefits of hyperparameter tuning for deep learning-based STDG models while also emphasizing the complexity it introduces, to include the performance assessment and comparison of the synthetic tabular data generated with the same STDG model but different parameter setting. This functionality can be added to the evaluation framework to identify the best parameter configuration of a STDG model to maximize the fidelity, utility and privacy results of the generated synthetic tabular data.

Regarding privacy guarantees of the STDG models, the analysis of the impact of DP on the different STDG models should be further explored with different privacy constraints. As the main focus of the paper was the presentation of the evaluation framework, the STDG models with privacy guarantees were trained using a single privacy budget value (ϵ=1.0), thus, the impact of the tradeoff metric ($G_{\epsilon }$) for varying noise levels was not analysed. Future work should explore the effects of adding DP to the STDG models with different ϵ values to better understand and provide a more extensive analysis of how varying levels of privacy can impact the STDG models’ performance, especially, on the tradeoff metric. Additionally, the incorporation of alternative privacy mechanisms beyond DP, such as the private aggregation of teacher ensembles (PATE) or secure multiparty computation (SMPC), to the STDG models should be investigated to determine and analyse how each one can affect the evaluation results of the generated synthetic tabular data.

Apart from that, the obtained results revealed specific improvement areas for some STDG models. For example, DP-TabDif consistently performed poorly in fidelity, utility, and tradeoff metrics across most datasets but excelled in privacy for two out of three datasets. A deeper analysis is required to identify the factors contributing to these results and to explore potential strategies to improve the fidelity of DP-TabDif while maintaining its privacy advantages. Similarly, NPC and DP-NPC demonstrated strong performance in fidelity, utility, and tradeoff metrics but struggled in privacy. Future studies should focus on enhancing the privacy capabilities of these models without sacrificing fidelity and utility.

Beyond these limitations and future work tasks, the proposed evaluation framework could be applied to other medical datasets with diverse and varying characteristics such as genomics data, signals, multimodal data, or time-series data. It can also be applied to other application domains, including industry, mobility and finances. Furthermore, evaluating purely numerical or categorical datasets, as well as datasets obtained directly from hospitals or laboratories, would further test the versatility and efficiency of the evaluation framework. Finally, the application of the framework could also be extended to federated environments to enable a performance evaluation in distributed systems. Collaborating with clinical experts to evaluate the clinical utility of the synthetic data generated would also provide critical insights into its real-world applicability.

## Data Availability

Publicly available datasets were analyzed in this study. This data can be found here: Acute Myeloid Leukemia dataset is available in Zenodo at https://zenodo.org/records/6878209. Brain Stroke dataset is available in Mendeley Data at https://data.mendeley.com/datasets/x8ygrw87jw/1. Cardiovascular Disease dataset is available in Kaggle at https://www.kaggle.com/datasets/sulianova/cardiovascular-disease-dataset.
