# Supplementary material for: Comprehensive evaluation framework for synthetic tabular data in health: fidelity, utility and privacy analysis of generative models with and without privacy guarantees
Source: Front Digit Health. 2025 Apr 24;7:1576290. doi: 10.3389/fdgth.2025.1576290 (PMC12058740; doi:10.3389/fdgth.2025.1576290)
Supplement: Supplementary file 1 [file Presentation1.pdf]

Supplementary Material: Detailed results and figures

This supplementary material provides extended results and visualisations for the evaluation framework applied to the three datasets (Acute Myeloid Leukemia, Brain Stroke, and Cardiovascular Disease). For each dataset, results for fidelity, utility, tradeoff, and privacy metrics are detailed, including relevant figures and tables to complement the main text.

1 ACUTE MYELOID LEUKEMIA DATASET

The Acute Myeloid Leukemia dataset [1] contains metadata and medical biomarkers from 1,540 patients diagnosed with Acute Myeloid Leukemia (AML). These patients participated in three prospective medical trials conducted by the German-Austrian AML Study Group (AMLSG). The dataset includes 12 variables: 6 numerical and 6 categorical attributes. Table S1 gathers the attributes of the dataset with the mean and standard deviation (std) values (for numerical) and mode and categories (for categorical).

Table S1. Attributes description for the AML dataset

Attribute name	Attribute type	Mean (±std)	Categories (mode)
Age	Numerical	48.42(±10.29)	-
Gender	Categorical	-	0,1 (1)
BM_Blasts	Numerical	67.47(±24.15)	-
HB	Numerical	9.15(±1.96)	-
perf_status	Numerical	0.89(±0.6)	-
PLT	Numerical	81.53(±86.34)	-
WBC	Numerical	36.01(±51.55)	-
secondary	Categorical	-	1,2,3 (1)
ahd	Categorical	-	0,1 (1)
OS	Numerical	3.28(±3.09)	-
OS_Status	Categorical	-	0,1 (1)
eln_2017	Categorical	-	favorable, adverse, intermediate (favorable)

1.1 Fidelity results

Figure S1 illustrates the mean Hellinger distances between real and synthetic data for each attribute across all STDG models, averaged over the 10 synthetic tabular data folds. The lowest distances were achieved by NPC (0.2193) and GC (0.2124), while the highest were observed for TabDif (0.3104), DP-TVAE (0.3010), and DP-GC (0.3006). The figure highlights that adding DP generally increases the Hellinger distance for most models (except DP-TabDif), indicating greater divergence from real data. Nevertheless, all distances

[1] https://www.nejm.org/doi/full/10.1056/NEJMoa1516192

remain below 0.8, suggesting that univariate distributions of synthetic attributes are reasonably similar to those of the real data.

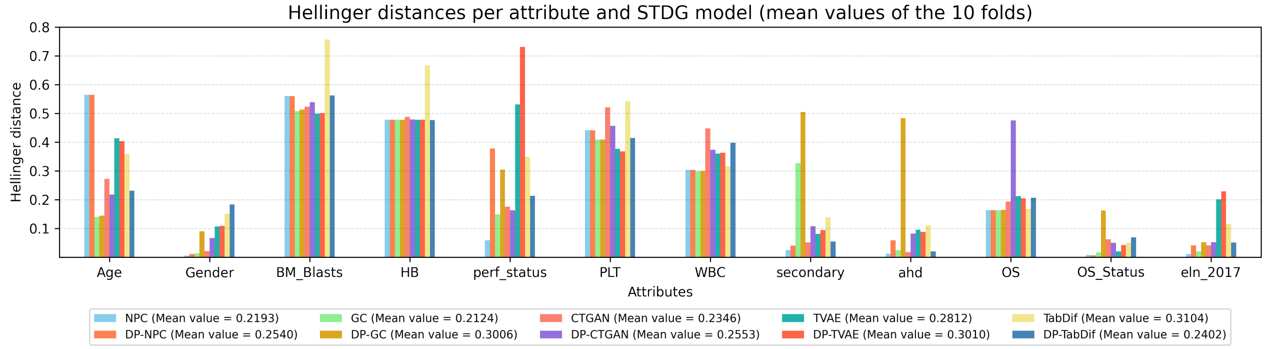

Figure S1. Hellinger distances per attributes and STDG models for Acute Myeloid Leukemia dataset

Figure S2 presents the pairwise correlation matrix of real data alongside the averaged correlation matrices from the 10 synthetic tabular data folds for each STDG model. Each synthetic matrix includes the corresponding PCD value and a p-value from a paired t-test to assess the statistical significance of differences between synthetic and real correlations. The figure shows that all models maintained correlations reasonably well, with PCD values below 0.25. Among the models, DP-GC was the only model to achieve no statistically significant difference ($p > 0.05$) and a PCD of 0.1563. NPC (0.0624) and DP-NPC (0.0628) exhibited the most visually similar matrices with the lowest PCD values. In contrast, CTGAN and DP-CTGAN showed the least similarity visually, though their PCD values remained low. Apart from that, DP-TVAE and DP-TabDif improved correlation preservation compared to their non-DP counterparts, while adding DP to other models did not visually impact correlation preservation. Overall, most models generated synthetic data with pairwise correlations closely resembling those of the real data, despite statistical differences ($p < 0.05$) in most cases.

Figure S3 presents the mean DD plots across 10 folds, comparing the depth measurements of synthetic tabular data generated by each STDG model with those of real tabular data. Each plot includes the R^2 metric, which indicates the proportion of variance in the synthetic depths explained by real depths. The best depth fits were obtained with NPC ($R^2 = 0.9975$) and DP-CTGAN ($R^2 = 0.9881$), while the poorest fits were observed for TabDif ($R^2 = 0.5534$), TVAE ($R^2 = 0.6873$) and DP-TVAE ($R^2 = 0.6823$). The remaining STDG models showed good depth alignment with R^2 values higher than 0.9. Furthermore, adding DP improved this metric for DP-GC, DP-CTGAN and DP-TabDif.

Figure S4 illustrates the mean ROC curves with the AUC of the Random Forest classifier trained to differentiate between real and synthetic samples across 10 folds for each STDG model. NPC ($AUC - ROC = 0.6568$), DP-NPC ($AUC - ROC = 0.7418$) and GC ($AUC - ROC = 0.7505$) were the only models approaching the ideal AUC-ROC of 0.5, indicating that the synthetic tabular data generated by these models was the least distinguishable from real data. In contrast, all other models showed higher AUC-ROC values, close to 1. Additionally, adding DP generally increased AUC-ROC score for most models, except DP-TabDif, suggesting that DP often reduces the distinguishability of synthetic samples from real ones.

Figure S5 summarises the fidelity results, displaying the mean, minimum, and maximum values across the 10 evaluation folds for each fidelity metric and STDG model, along with the associated p-value for comparison between model pairs (base and DP counterpart). This figure visually supplements Table 3 and

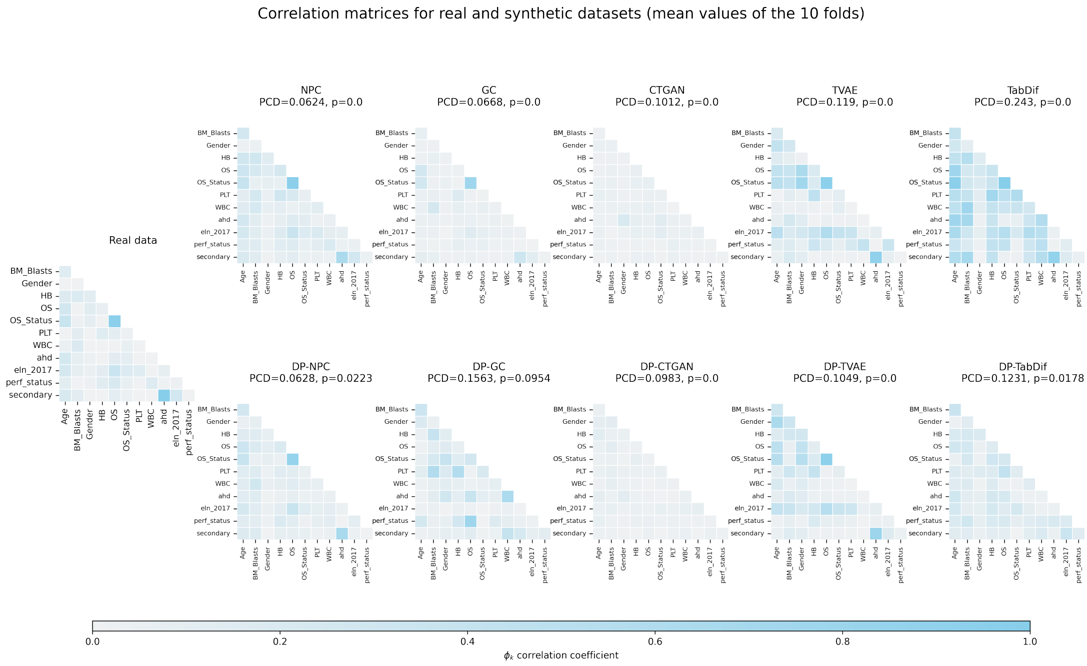

Figure S2. Correlation matrices of real data and synthetic data generated by each STDG model for Acute Myeloid Leukemia dataset

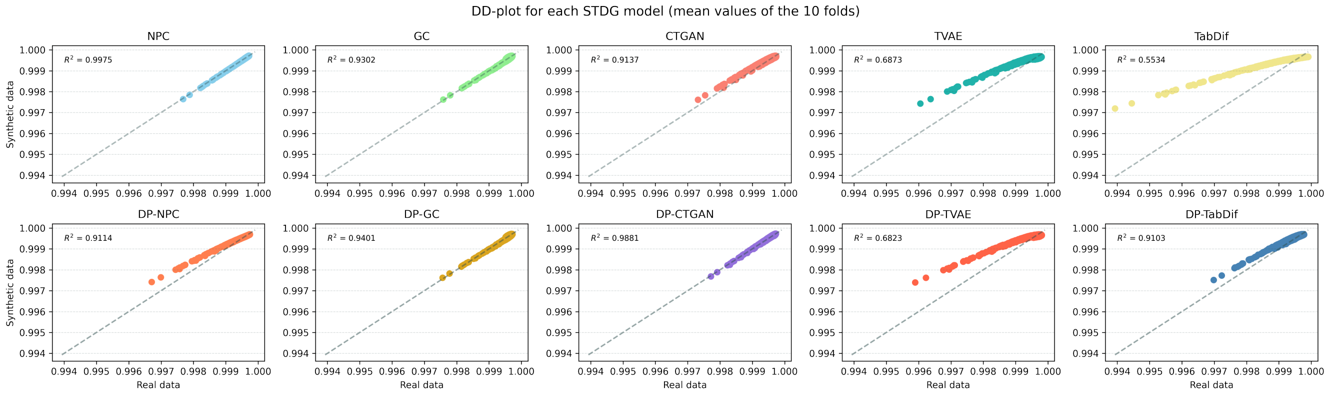

Figure S3. DDplot of real and synthetic data adjustments per each STDG model for Acute Myeloid Leukemia dataset

Section 3.1.1 of the main text, providing a clear representation of the variability and significance of fidelity metrics across models.

1.2 Utility results

Figure S6 illustrates the mean distributions of the classification metrics across the 10 folds for TRTR and TSTR with each STDG model. The plots include the associated p-values from a paired t-test comparing TRTR and TSTR distributions, as well as the mean TRTR-TSTR metric differences (δ) to highlight significant differences between classification metrics distributions. For NPC, DP-NPC, TVAE, DP-TVAE and TabDif, classification metric distributions when TSTR showed no significant difference ($p > 0.05$) compared to TRTR, with low metric differences ($\delta < 0.1$). The remaining STDG models showed statistically significant differences ($p < 0.05$) in classification metrics distributions compared to TRTR, but

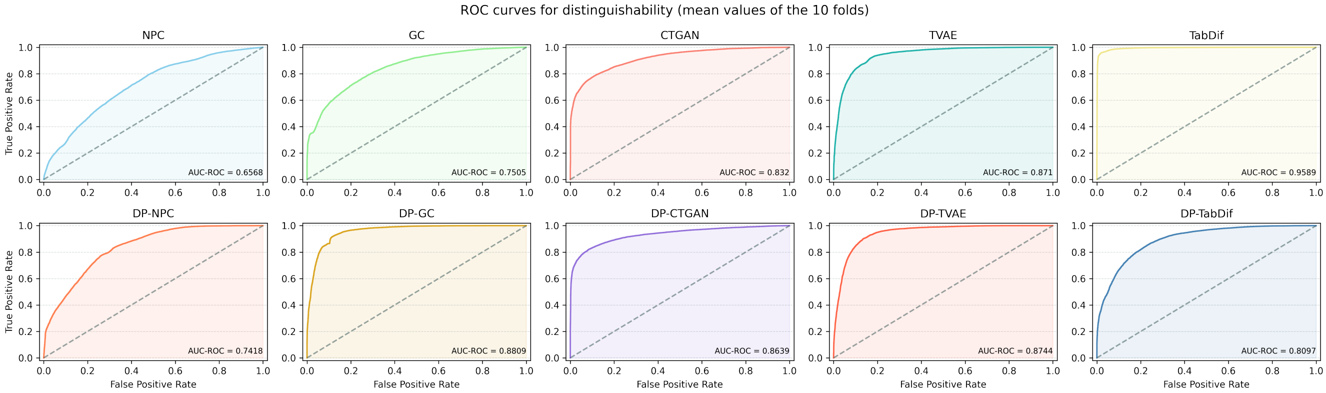

Figure S4. Distinguishability ROC curves of synthetic tabular data generated by each STDG model for Acute Myeloid Leukemia dataset

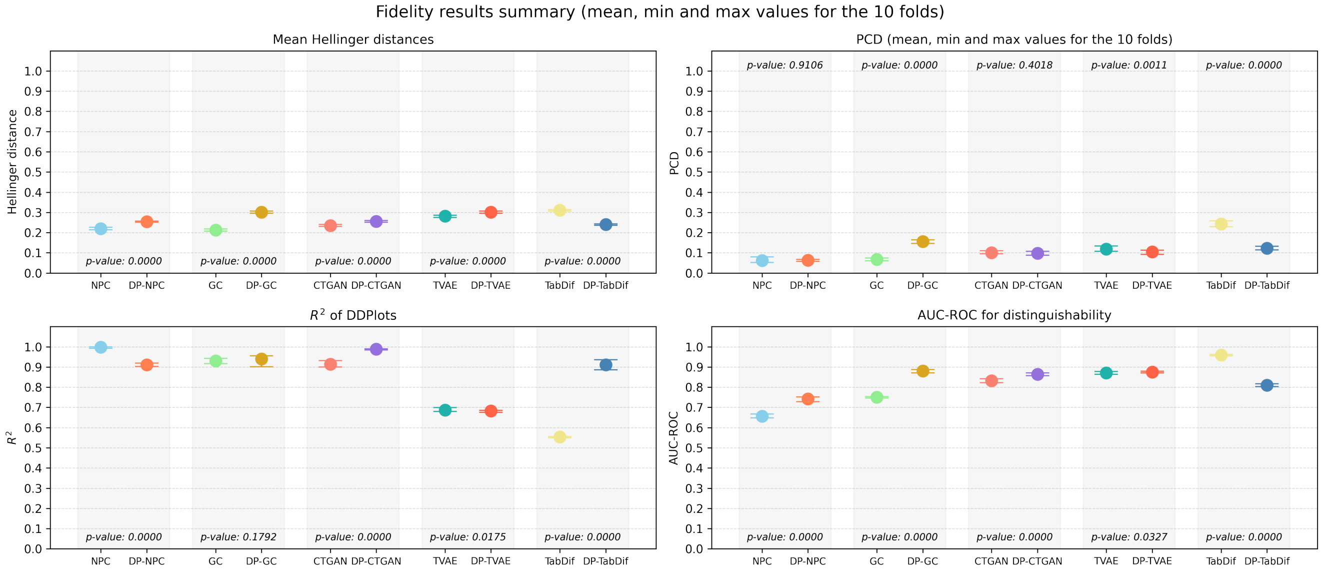

Figure S5. Fidelity results summary over the 10 folds for the synthetic tabular data generated by each STDG model for Acute Myeloid Leukemia dataset.

still maintained low classification metric differences ($\delta < 0.2$). These results suggest that synthetic tabular data retains good utility for classification tasks across all models, despite some significant differences in some metrics.

Figure S7 illustrates the classification metrics differences when TRTR and TSTR are computed, displaying the mean, minimum, and maximum metric differences across the 10 evaluation folds for each classification metric and STDG model, along with the associated p-value for comparison between model pairs (base and DP counterpart). This figure visually supplements Table 4 and Section 3.1.2 of the main text, providing a clear representation of the variability and significance of classification metrics differences across models.

Figure S8 illustrates the mean distributions of the regression metrics across the 10 folds for TRTR and TSTR with each STDG model. The plots include the associated p-values from a paired t-test comparing TRTR and TSTR distributions, as well as the mean TRTR-TSTR metric differences (δ) to highlight significant differences between regression metrics distributions. Regression metric distributions for all

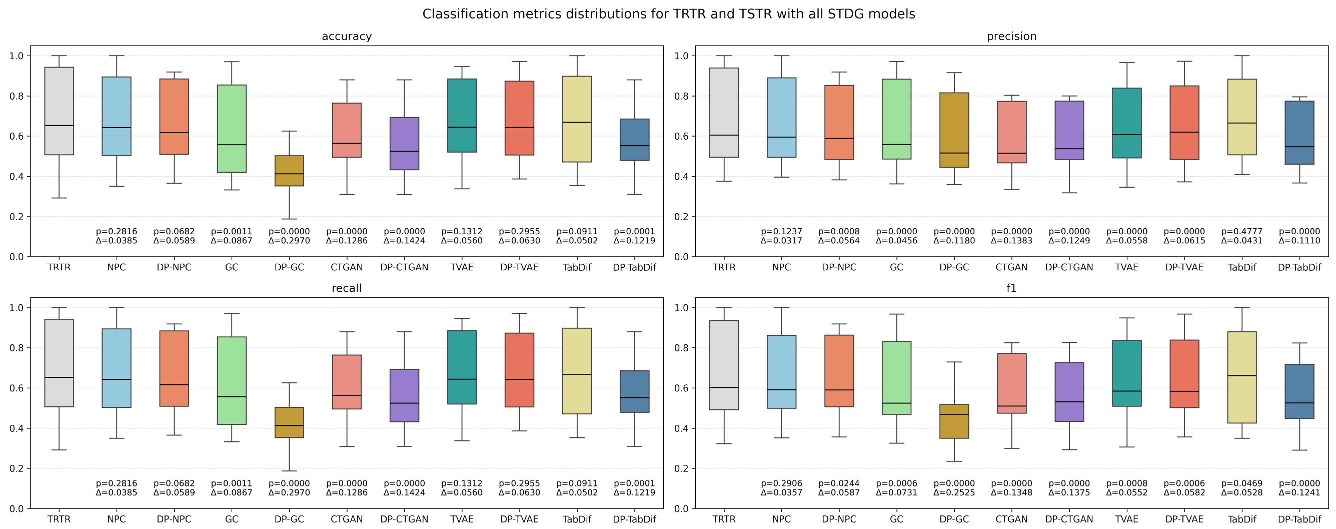

Figure S6. TRTR and TSTR results of classification metrics for Acute Myeloid Leukemia dataset.

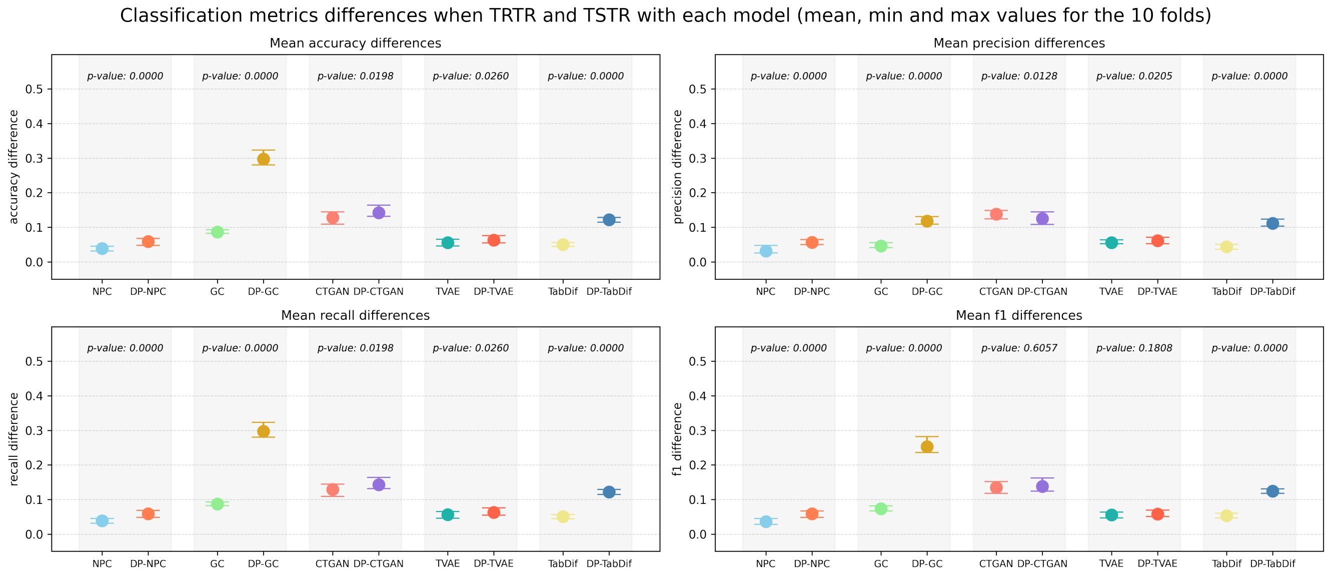

Figure S7. Classification differences between TRTR and TSTR results summay over the 10 folds for the synthetic tabular data generated by each STDG model for Acute Myeloid Leukemia dataset.

models showed statistically significant differences ($p < 0.05$) compared to TRTR, but with low metric differences ($\Delta < 0.2$) in most cases. An exception was TabDif, which exhibited the higher metric differences. These results suggest that synthetic tabular data maintains good utility for regression tasks across all models except TabDif, despite significant differences in all metrics compared to TRTR.

Figure S9 summarises the regression metrics differences when TRTR and TSTR are computed, displaying the mean, minimum, and maximum metric differences across the 10 evaluation folds for each regression metric and STDG model, along with the associated p-value for comparison between model pairs (base and DP counterpart). This figure visually supplements Table 4 and Section 3.1.2 of the main text, providing a clear representation of the variability and significance of regression metrics differences across models.

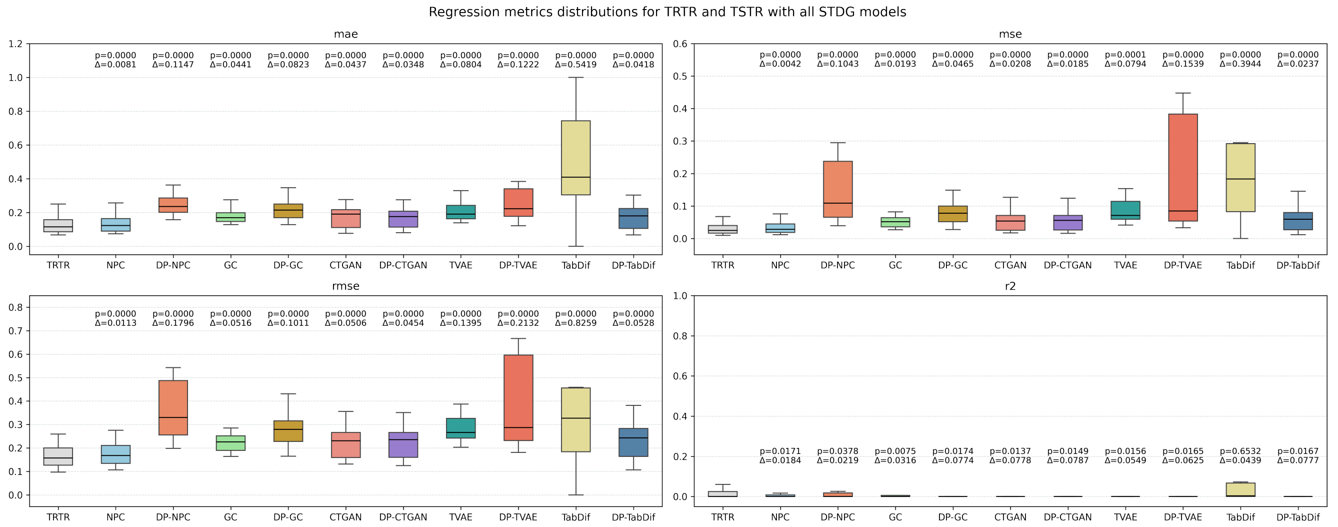

Figure S8. TRTR and TSTR results of regression metrics for Acute Myeloid Leukemia dataset.

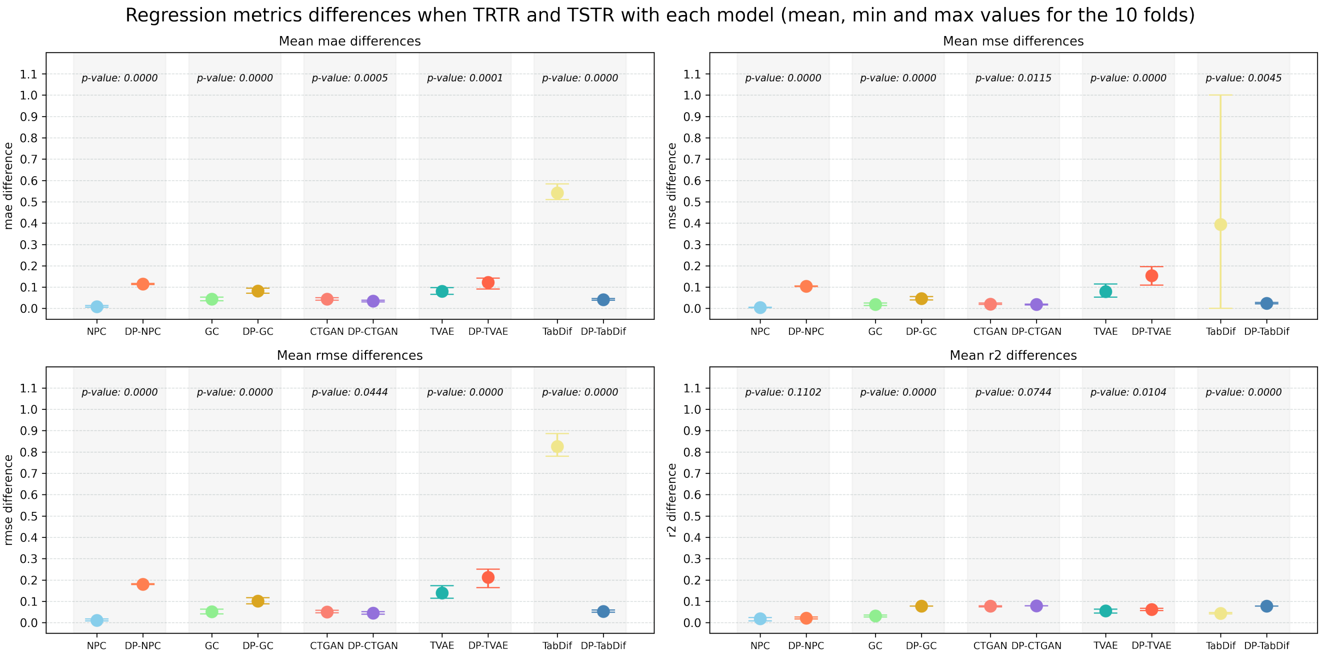

Figure S9. Regression differences between TRTR and TSTR results summay over the 10 folds for the synthetic tabular data generated by each STDG model for Acute Myeloid Leukemia dataset.

1.3 Tradeoff results

Figure S10 summarises the fidelity-utility tradeoff results, displaying the mean, minimum, and maximum values of the G metric across the 10 evaluation folds for each STDG model, along with the associated p-value for comparison between model pairs (base and DP counterpart). This figure visually supplements tradeoff results of Table 3 and Section 3.1.1 of the main text, providing a clear representation of the variability and significance of the G metric across STDG models.

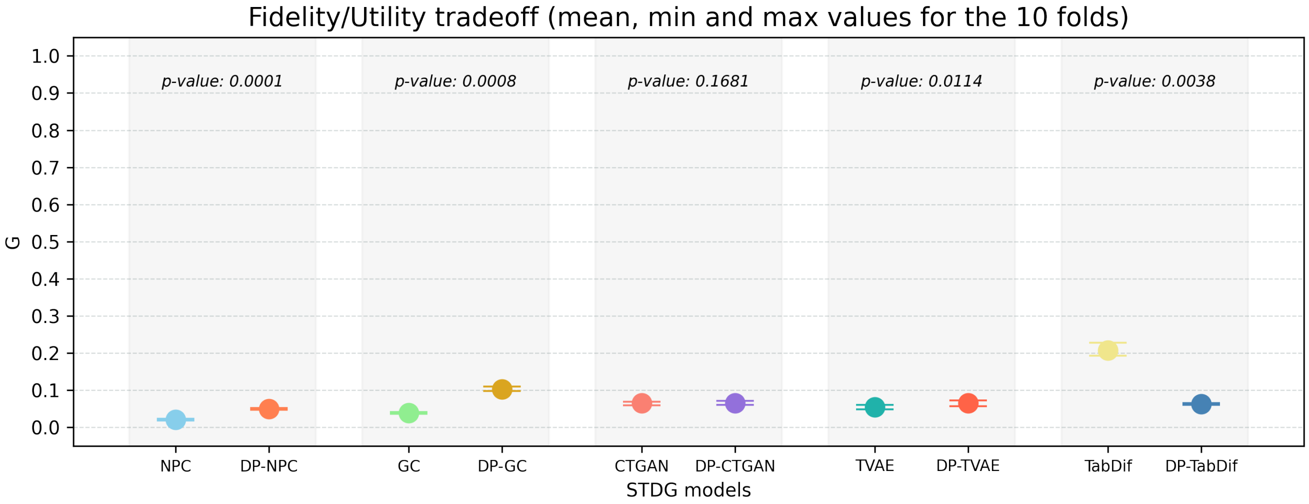

Figure S10. Tradeoff results summary over the 10 folds for the synthetic tabular data generated by each STDG model for Acute Myeloid Leukemia dataset.

1.4 Privacy attacks results

Figure S11 illustrates the attack risks with confidence intervals, averaged across the 10 synthetic tabular data folds for each STDG model and for linkability, univariate singlingout, multivariate singlingout and membership inference attacks. Linkability and univariate singlingout attack risks were very low across all models, remaining close to 0 and below 0.1. Multivariate singlingout attack risks were slightly higher, with NPC exhibiting the highest risk (0.2) and TabDif the lowest (0.0), while risks for the other models ranged between 0.05 and 0.2. Membership inference attack risk was highest for NPC (0.3), significantly exceeding the risks observed for other models, which remained below 0.05. These results suggest that synthetic tabular data generated by all STDG models presents minimal privacy risks overall.

Figure S12 presents the attributes inference attack risks with confidence intervals, averaged across the 10 synthetic tabular data folds for each STDG model and dataset attributes. The higher risks were observed for NPC and DP-NPC across all attributes, with risk values ranging from 0.4 and 0.6. In contrast, the risks for all other models remained below 0.2 for every attribute. These findings indicate that NPC and DP-NPC performed the worst in protecting against attribute inference attacks, showing a likelihood of approximately 50% to be linked to real data attributes.

Figure S13 summarises the resulting risks for all privacy attacks, displaying the mean, minimum, and maximum risks across the 10 evaluation folds for each STDG model, along with the associated p-value for comparison between model pairs (base and DP counterpart). This figure visually supplements the privacy attack results of Table 5 and Section 3.1.3 of the main text, providing a clear representation of the variability and significance of the risks for privacy attacks across STDG models.

2 BRAIN STROKE DATASET

The Brain Stroke dataset [2] contains 38,962 records consisting of patient metadata and risk factors for predicting the likelihood of a brain stroke. It includes 17,711 attributes: 3 numerical and 8 categorical. Table

[2] https://data.mendeley.com/datasets/x8ygrw87jw/1

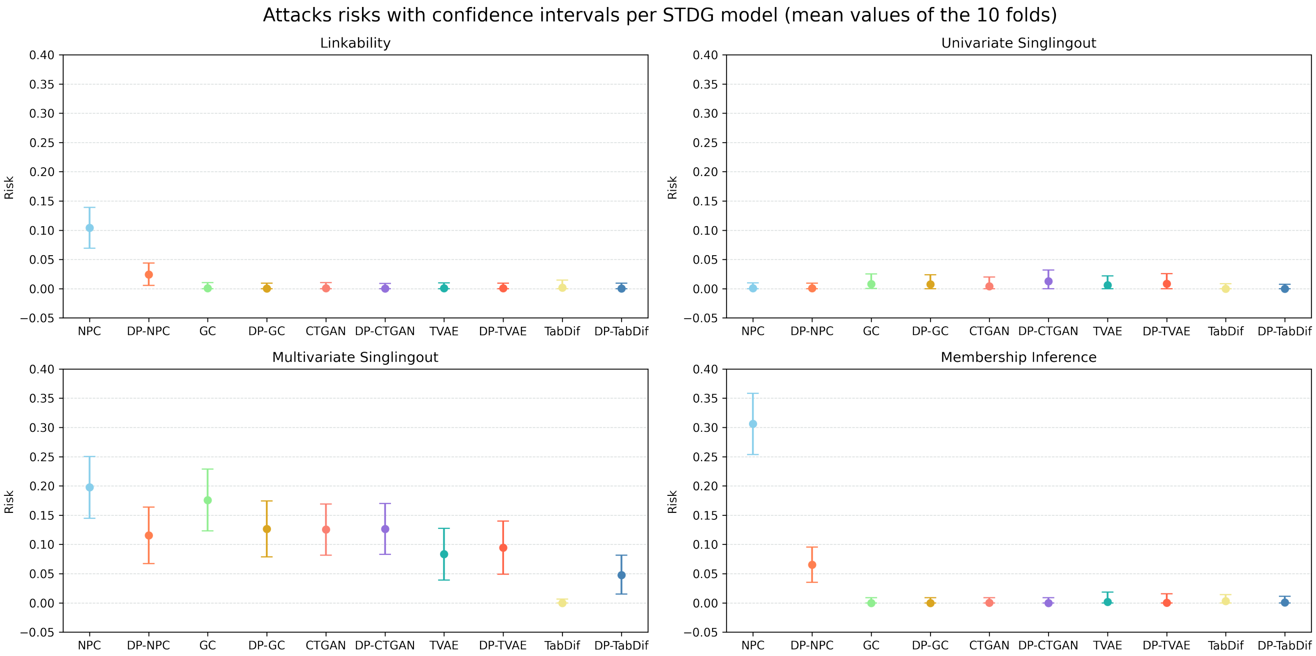

Figure S11. Attacks risks with confidence intervals for synthetic data generated by each STDG model for Acute Myeloid Leukemia dataset

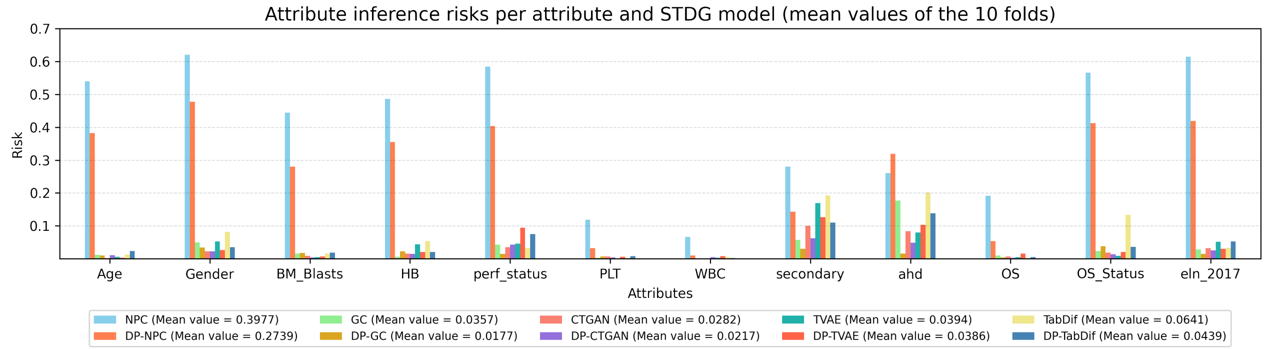

Figure S12. Attributes inference risks per attributes and STDG models for Acute Myeloid Leukemia dataset

S2 gathers the attributes of the dataset with the mean and standard deviation (std) values (for numerical) and mode and categories (for categorical).

2.1 Fidelity results

Figure S14 illustrates the mean Hellinger distances between real and synthetic data for each attribute across all STDG models, averaged over the 10 synthetic tabular data folds. The lowest distances were achieved by CTGAN (0.1365), NPC (0.1594) and GC (0.1615), while the highest were observed for DP-TabDif (0.3128), DP-TVAE, DP-GC (0.2835) and DP-TVAE (0.2408). The figure highlights that adding DP generally increases the Hellinger distance for most models, indicating greater divergence from real data. Nevertheless, all distances remain below 0.7, suggesting that univariate distributions of synthetic attributes are reasonably similar to those of the real data.

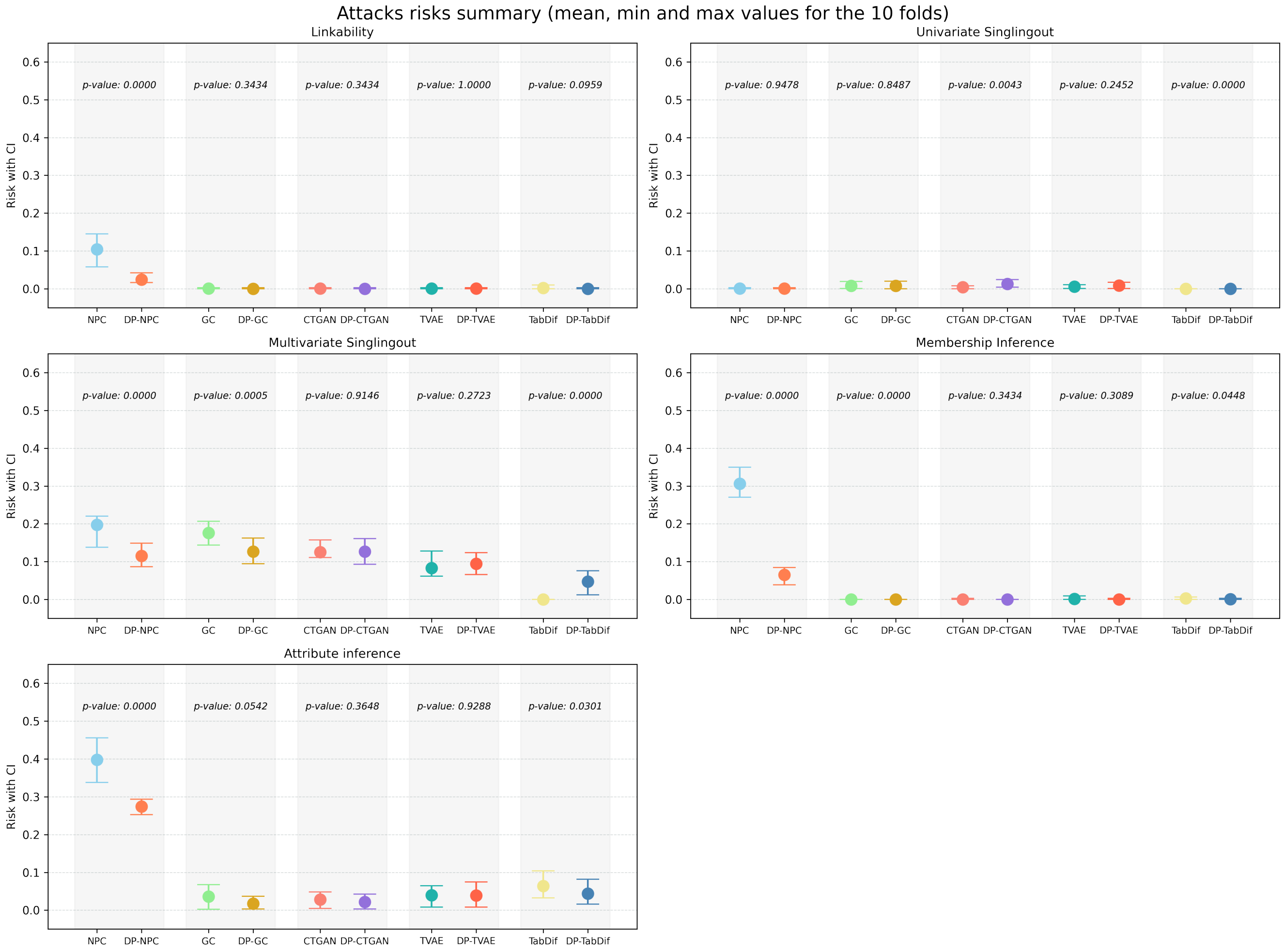

Figure S13. Attacks risks summary for synthetic data generated by each STDG model for Acute Myeloid Leukemia dataset

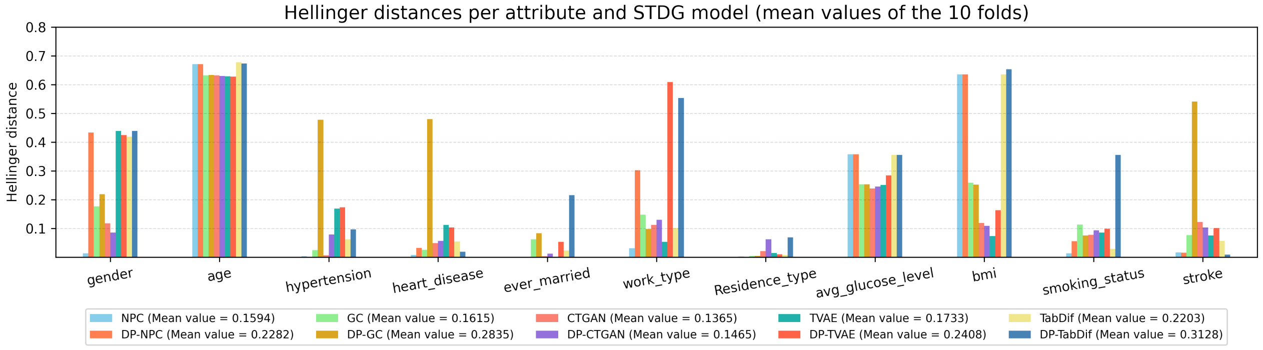

Figure S14. Hellinger distances per attributes and STDG models for Brain Stroke dataset.

Figure S15 presents the pairwise correlation matrix of real data alongside the averaged correlation matrices from the 10 synthetic tabular data folds for each STDG model. Each synthetic matrix includes the corresponding PCD value and a p-value from a paired t-test to assess the statistical significance of differences between synthetic and real correlations. The figure shows that all models maintained correlations

Table S2. Attributes description for the Brain Stroke dataset

Attribute name	Attribute type	Mean (±std)	Categories (mode)
gender	Categorical	-	Female, Male, Other (Female)
age	Numerical	$42.42(\pm 22.54)$	-
hypertension	Categorical	-	0,1 (0)
heart_disease	Categorical	-	0,1 (0)
ever_married	Categorical	-	Yes, No (Yes)
work_type	Categorical	-	Private, Self-employed, children, Govt_job, Never_worked (Private)
Residence_type	Categorical	-	Urban, Rural (Urban)
avg_glucose_level	Numerical	$104.77(\pm 43.51)$	-
bmi	Numerical	$28.62(\pm 7.68)$	-
smoking_status	Categorical	-	never smoked, formerly smoked, smokes, Unknown (never smoked)
stroke	Categorical	-	0,1 (0)

reasonably well, with PCD values below 0.21. Among the models, TVAE and DP-TabDif were the only models to achieve no statistically significant difference ($p > 0.05$) correlation matrix compared to real data correlation matrix with PCD of 0.1065 and 0.3691, respectively. NPC ($PCD = 0.012$), DP-NPC ($PCD = 0.0401$) and DP-CTGAN ($PCD = 0.0726$) exhibited the most visually similar matrices with the lowest PCD values. In contrast, GC, DP-GC, TVAE, DP-TVAE and TabDif showed the least similarity visually, though their PCD values remained low. Apart from that, adding DP to the models did not visually impact correlation preservation quite much.

Figure S16 presents the mean DD plots across 10 folds, comparing the depth measurements of synthetic tabular data generated by each STDG model with those of real tabular data. Each plot includes the R^2 metric, which indicates the proportion of variance in the synthetic depths explained by real depths. All models except TabDif and DP-TabDiff showed good depth alignment with R^2 values higher or around 0.9. The poorer fit was obtained with DP-TabDif, with a R^2 of 0.4877. Overall, adding DP did not impact this metric for DP-NPC, DP-GC and DP-CTGAN. For, DP-TVAE and DP-TabDif, R^2 values were reduced compared to their non-DP counterpart.

Figure S17 illustrates the mean ROC curves with the AUC of the Random Forest classifier trained to differentiate between real and synthetic samples across 10 folds for each STDG model. NPC ($AUC - ROC = 0.5333$), DP-NPC ($AUC - ROC = 0.5752$), CTGAN ($AUC - ROC = 0.6195$) and DP-CTGAN (($AUC - ROC = 0.6385$) were the only models approaching the ideal AUC-ROC of 0.5, indicating that the synthetic tabular data generated by these models was the least distinguishable from real data. In contrast, all other models showed higher AUC-ROC values, close to 1. Additionally, adding DP generally increased AUC-ROC score for most models, suggesting that DP maximises the distinguishability of synthetic samples from real ones.

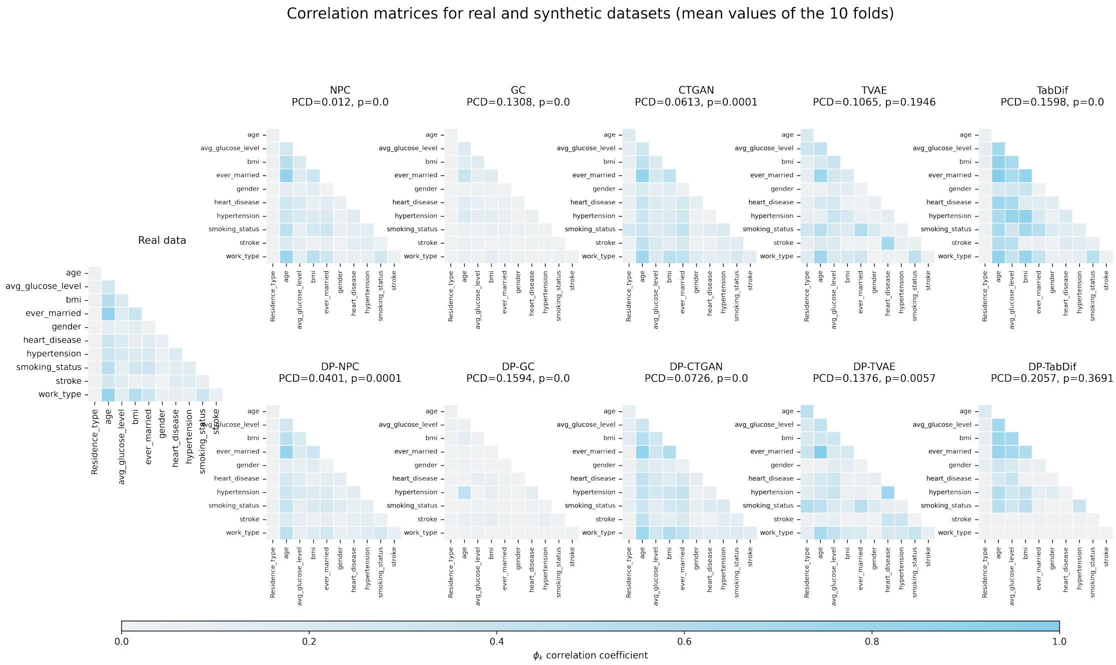

Figure S15. Correlation matrices of real data and synthetic data generated by each STDG model for Brain Stroke dataset.

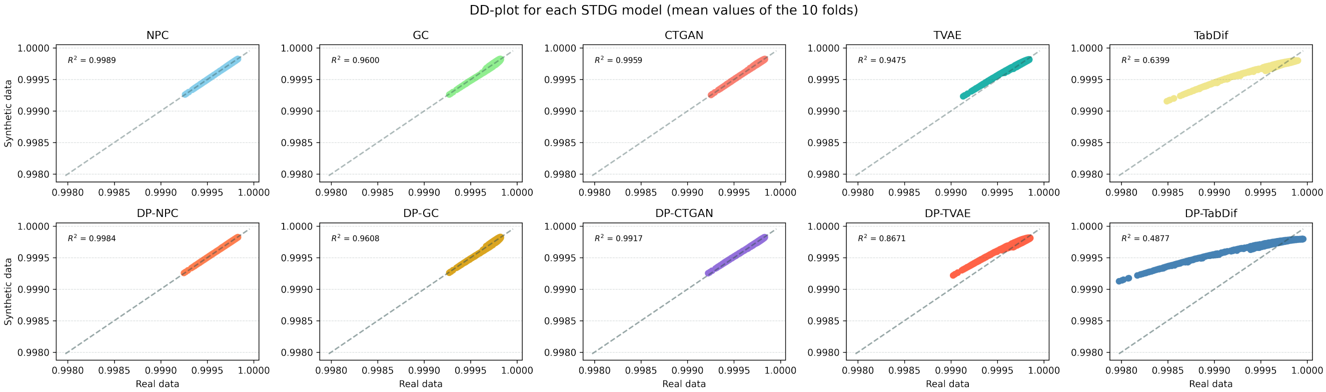

Figure S16. DDplot of real and synthetic data adjustments per each STDG model for Brain Stroke dataset.

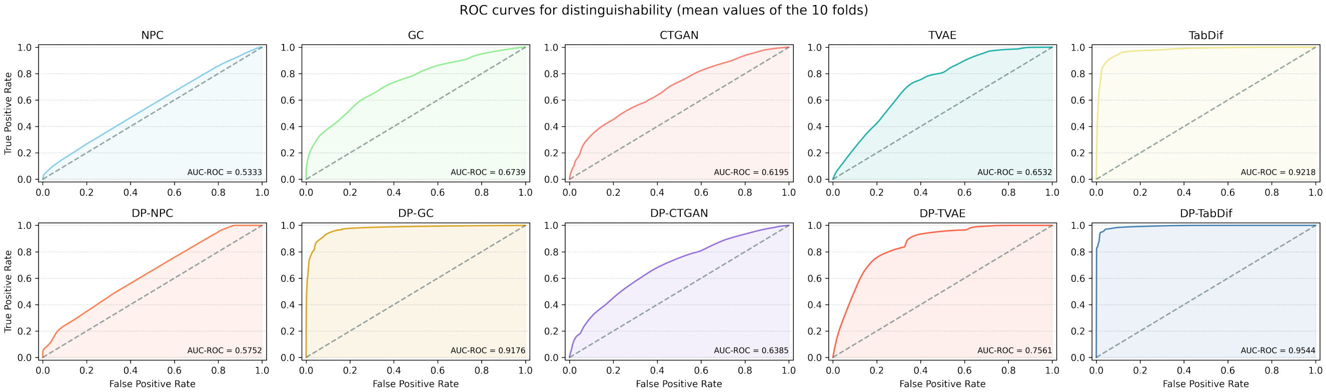

Figure S17. Distinguishability ROC curves of synthetic tabular data generated by each STDG model for Brain Stroke dataset.

Figure S18 summarises the fidelity results, displaying the mean, minimum, and maximum values across the 10 evaluation folds for each fidelity metric and STDG model, along with the associated p-value for comparison between model pairs (base and DP counterpart). This figure visually supplements Table 6 and Section 3.2.1 of the main text, providing a clear representation of the variability and significance of fidelity metrics across models.

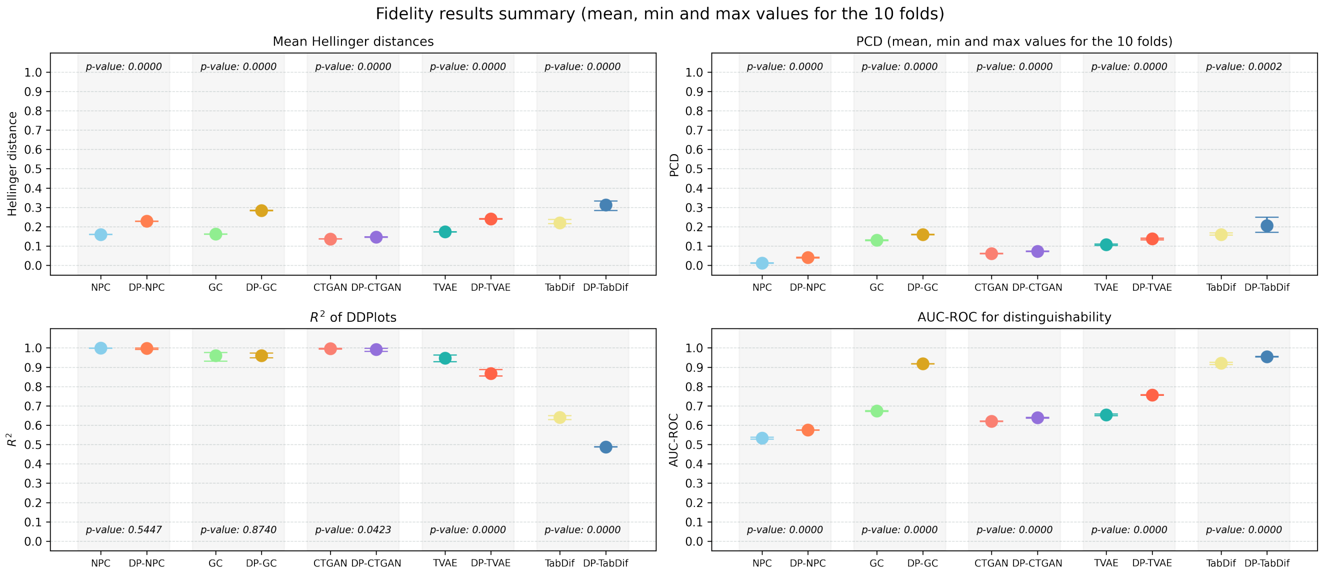

Figure S18. Fidelity results summary over the 10 folds for the synthetic tabular data generated by each STDG model for Brain Stroke dataset.

2.2 Utility results

Figure S19 illustrates the mean distributions of the classification metrics across the 10 folds for TRTR and TSTR with each STDG model. The plots include the associated p-values from a paired t-test comparing TRTR and TSTR distributions, as well as the mean TRTR-TSTR metric differences (Δ) to highlight significant differences between classification metrics distributions. All STDG models showed statistically significant differences ($p < 0.05$) in classification metrics distributions compared to TRTR, but still maintained low classification metric differences ($\delta < 0.24$), being the higher ones for TabDif and DP-TabDif, ranging from 0.18 to 0.24. These results suggest that synthetic tabular data retains good utility for classification tasks across all models, despite all metrics distributions of TSTR for all models being significantly different compared to TRTR.

Figure S20 displays the classification metrics differences when TRTR and TSTR, displaying the mean, minimum, and maximum metric differences across the 10 evaluation folds for each classification metric and STDG model, along with the associated p-value for comparison between model pairs (base and DP counterpart). This figure visually supplements Table 7 and Section 3.2.2 of the main text, providing a clear representation of the variability and significance of classification metrics differences across models.

Figure S21 illustrates the mean distributions of the regression metrics across the 10 folds for TRTR and TSTR with each STDG model. The plots include the associated p-values from a paired t-test comparing TRTR and TSTR distributions, as well as the mean TRTR-TSTR metric differences (Δ) to highlight significant differences between regression metrics distributions. All STDG models showed statistically significant differences ($p < 0.05$) in regression metrics distributions compared to TRTR, but still maintained

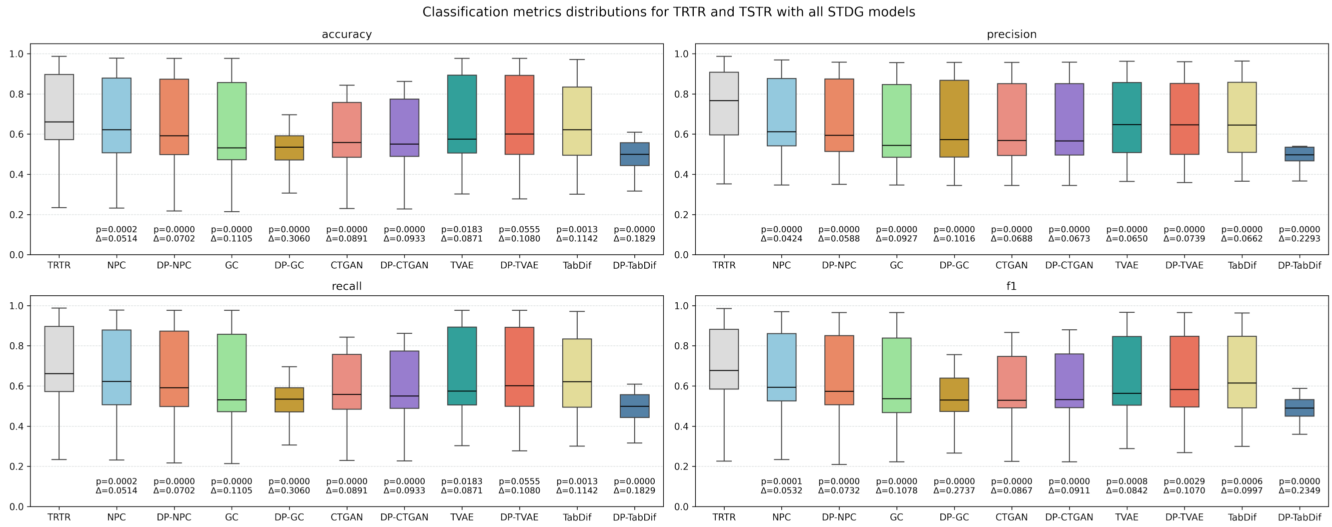

Figure S19. TRTR and TSTR results of classification metrics for Brain Stroke dataset

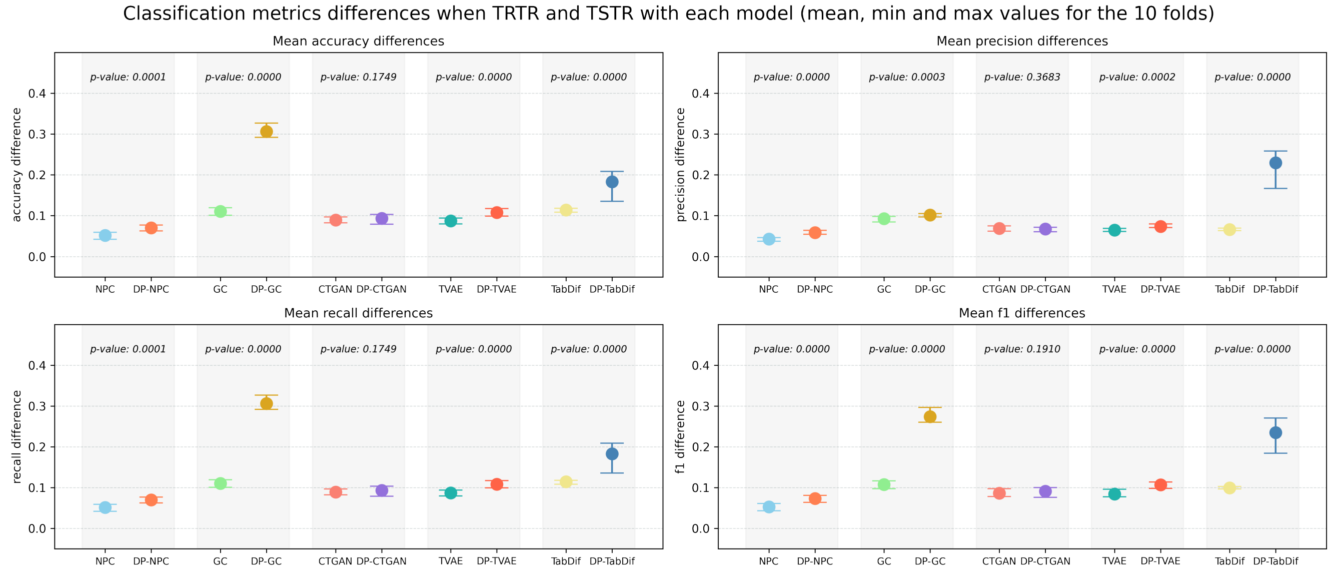

Figure S20. Classification differences between TRTR and TSTR results summay over the 10 folds for the synthetic tabular data generated by each STDG model for Brain Stroke dataset.

low classification metric differences ($\delta < 0.1$) for all models expect TabDif and DP-TabDif ranging from 0.1 to 0.7. These results suggest that synthetic tabular data retains good utility for regression tasks across all models except TabDif and DP-TabDif, despite all metrics distributions of TSTR for all models being significantly different compared to TRTR.

Figure S22 summarises the regression metrics differences when TRTR and TSTR, displaying the mean, minimum, and maximum metric differences across the 10 evaluation folds for each regression metric and STDG model, along with the associated p-value for comparison between model pairs (base and DP counterpart). This figure visually supplements Table 7 and Section 3.2.2 of the main text, providing a clear representation of the variability and significance of regression metrics differences across models.

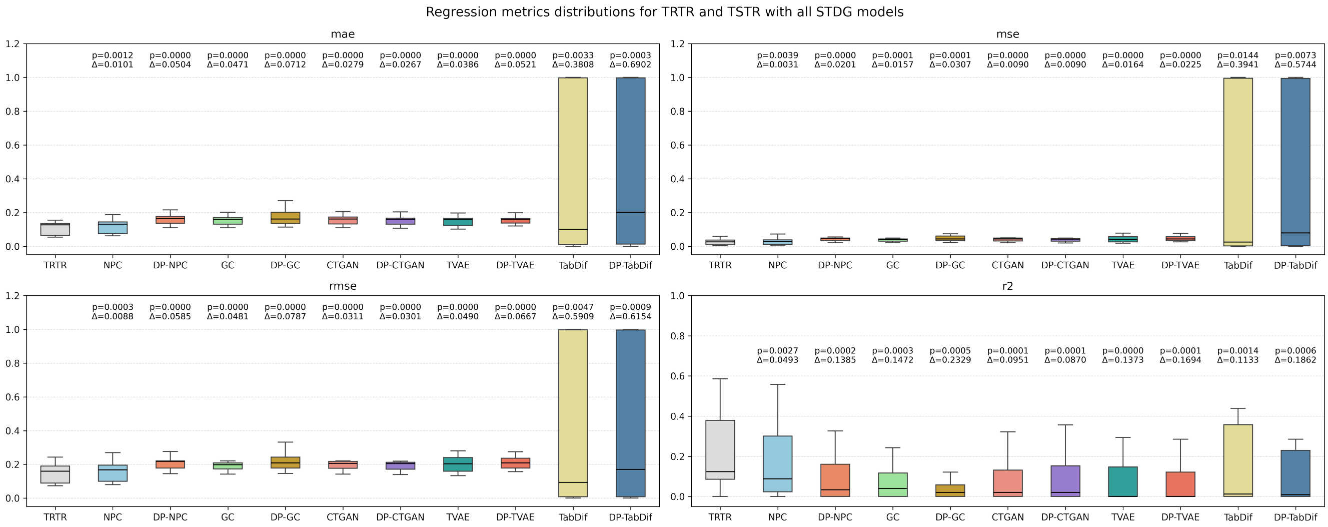

Figure S21. TRTR and TSTR results of regression metrics for Brain Stroke dataset.

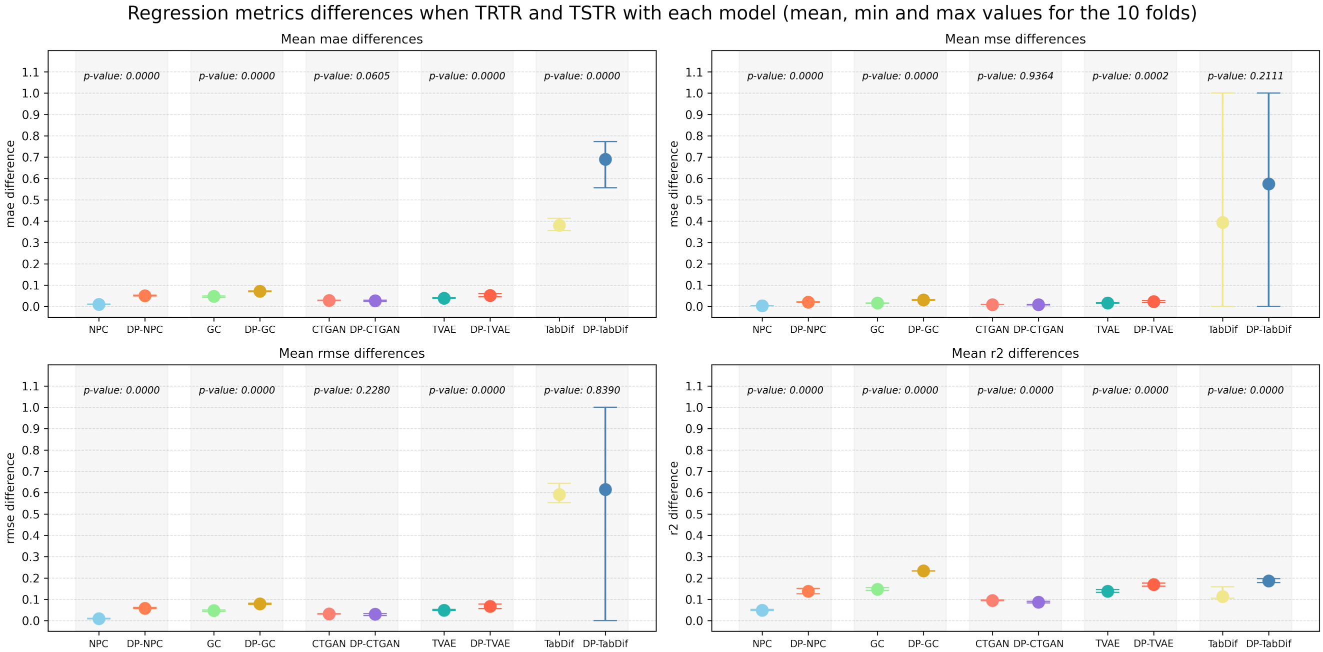

Figure S22. Regression differences between TRTR and TSTR results summay over the 10 folds for the synthetic tabular data generated by each STDG model for Brain Stroke dataset

2.3 Tradeoff results

Figure S23 summarises the fidelity-utility tradeoff results, displaying the mean, minimum, and maximum values of the G metric across the 10 evaluation folds for each STDG model, along with the associated p-value for comparison between model pairs (base and DP counterpart). This figure visually supplements tradeoff results of Table 6 and Section 3.2.1 of the main text, providing a clear representation of the variability and significance of the G metric across STDG models.

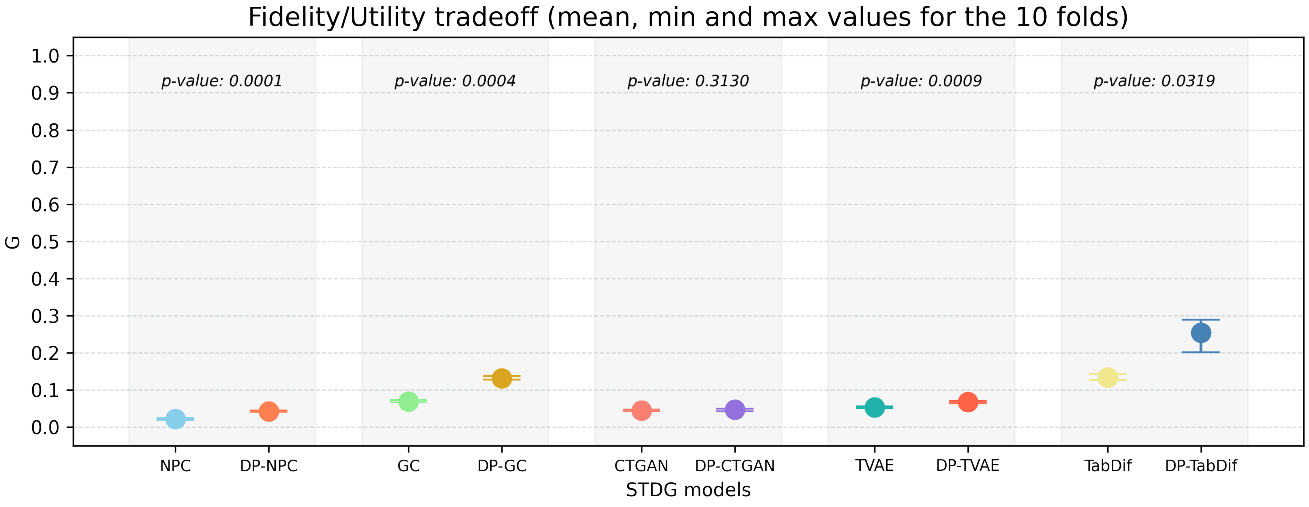

Figure S23. Tradeoff results summary over the 10 folds for the synthetic tabular data generated by each STDG model for Brain Stroke dataset

2.4 Privacy attacks results

Figure S24 illustrates the attack risks with confidence intervals, averaged across the 10 synthetic tabular data folds for each STDG model and for linkability, univariate singlingout, multivariate singlingout and membership inference attacks. Linkability, univariate singlingout and multivariate attack risks were very low across all models, being lower than 0.2. Higher membership inference attack risks were observed for NPC (0.55) and DP-NPC (0.3), exceeding the risks observed for other models, which remained below 0.1. These results suggest that synthetic tabular data generated by all STDG models presents minimal privacy risks overall, except for NPC and DP-NPC models.

Figure S25 presents the attributes inference attack risks with confidence intervals, averaged across the 10 synthetic tabular data folds for each STDG model and dataset attributes. The highest risks were observed for NPC and DP-NPC across all attributes, with risk values ranging from 0.2 and 0.4. In contrast, the risks for all other models remained below 0.1 for every attribute. These findings indicate that NPC and DP-NPC performed the worst in protecting against attribute inference attacks, with the attributes showing a likelihood of approximately 35% to be linked to real data attributes.

Figure S26 summarises the resulting risks for all privacy attacks, displaying the mean, minimum, and maximum risks across the 10 evaluation folds for each STDG model, along with the associated p-value for comparison between model pairs (non-DP and DP). This figure visually supplements the privacy attack results of Table 8 and Section 3.2.3 of the main text, providing a clear representation of the variability and significance of the risks for privacy attacks across STDG models.

3 CARDIOVASCULAR DISEASE DATASET

The Cardiovascular Disease dataset [3] contains 70,000 records with metadata and risk factors to predict the occurrence of cardiovascular disease. The dataset includes 13 attributes: 6 numerical and 7 categorical.

[3] https://www.kaggle.com/datasets/sulianova/cardiovascular-disease-dataset

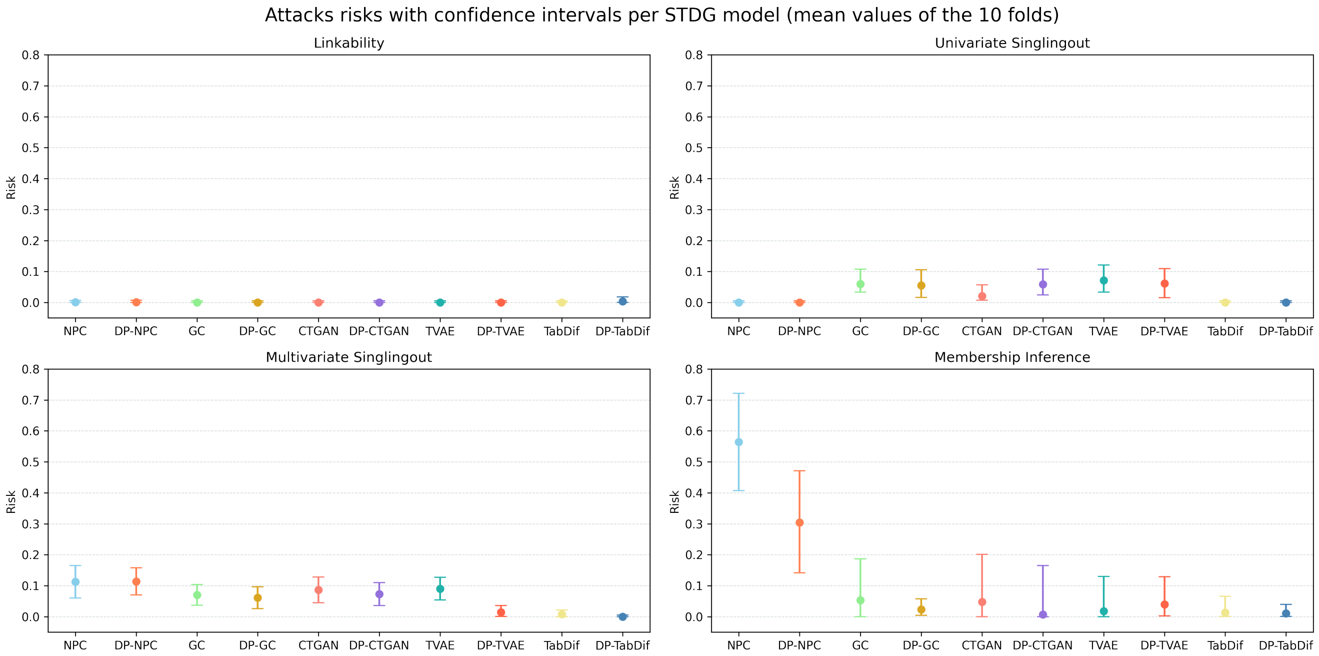

Figure S24. Attacks risks with confidence intervals for synthetic data generated by each STDG model for Brain Stroke dataset.

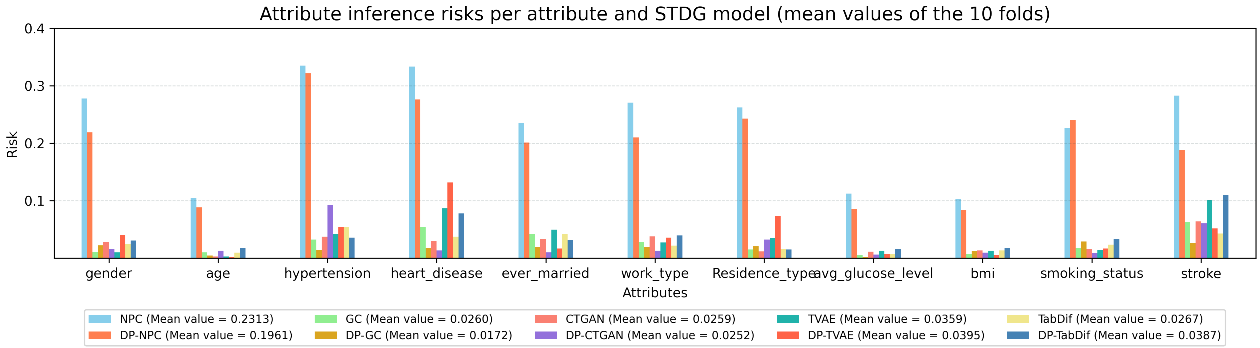

Figure S25. Attributes inference risks per attributes and STDG models for Brain Stroke dataset.

Table S3 gathers the attributes of the dataset with the mean and standard deviation (std) values (for numerical) and mode and categories (for categorical).

3.1 Fidelity results

Figure S27 illustrates the mean Hellinger distances between real and synthetic data for each attribute across all STDG models, averaged over the 10 synthetic tabular data folds. The lowest distances were achieved by NPC (0.2539), DP-NPC (0.2544) and GC (0.2779), while the highest were observed for DP-TabDif (0.4084), DP-GC (0.3975) and TabDif (0.3592). The figure highlights that adding DP generally increases the Hellinger distance for most models except DP-TVAE, indicating greater divergence from real data. Nevertheless, all distances remain below 0.5 for 9 out of 13 variables, suggesting that univariate distributions of synthetic attributes are reasonably similar to those of the real data for more than half of the variables.

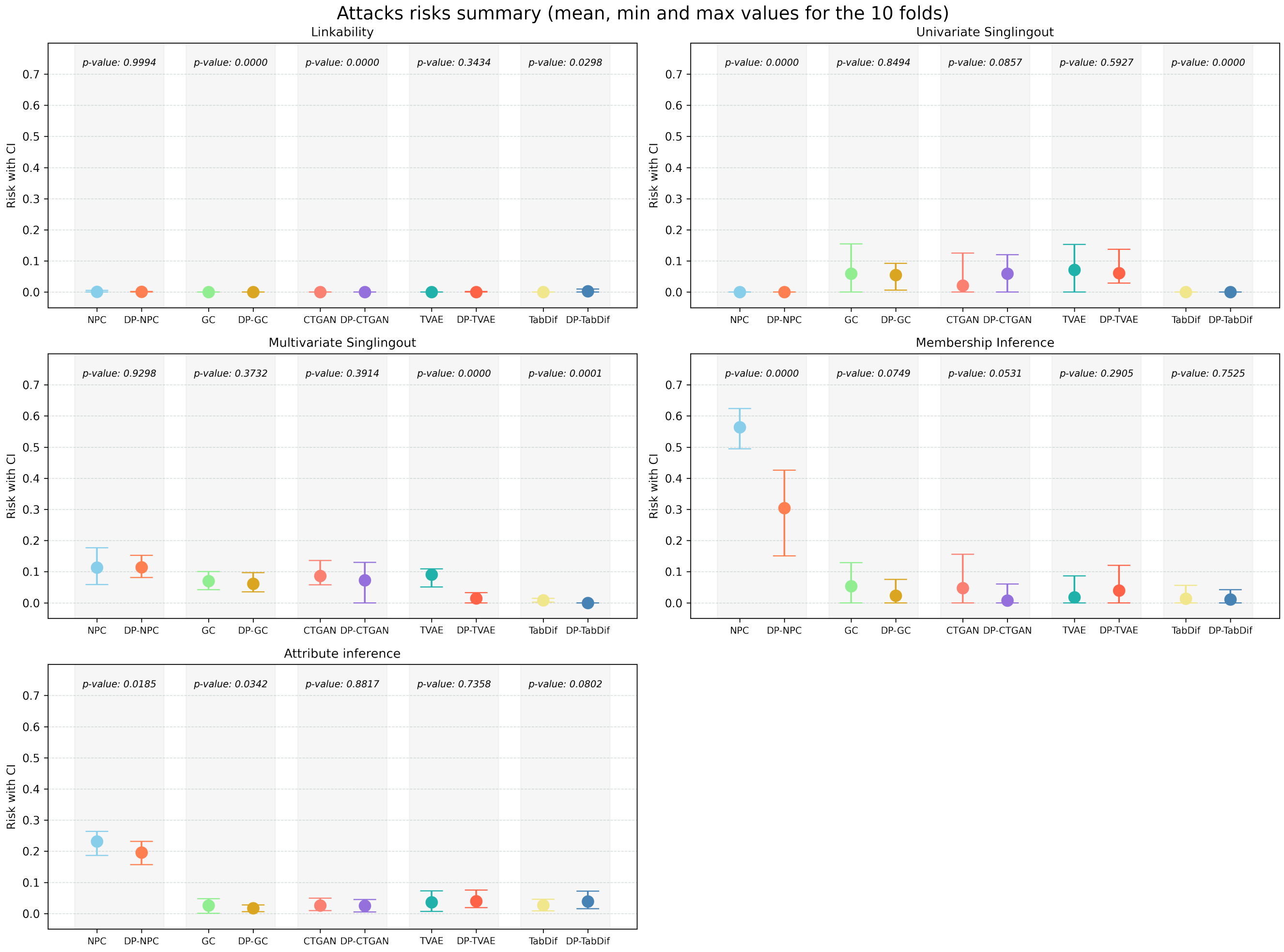

Figure S26. Attacks risks summary for synthetic data generated by each STDG model for Brain Stroke dataset.

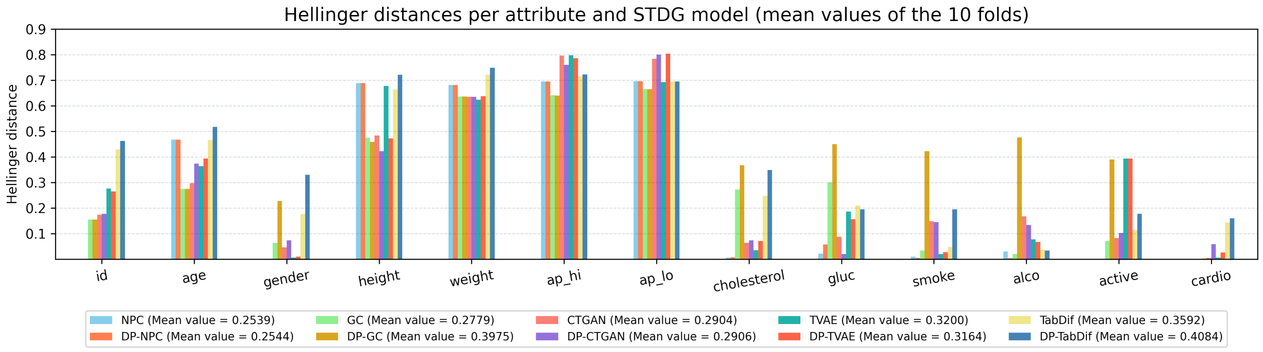

Figure S27. Hellinger distances per attributes and STDG models for Cardiovascular Disease dataset.

Figure S28 presents the pairwise correlation matrix of real data alongside the averaged correlation matrices from the 10 synthetic tabular data folds for each STDG model. Each synthetic matrix includes the corresponding PCD value and a p-value from a paired t-test to assess the statistical significance of differences between synthetic and real correlations. The figure shows that all models maintained correlations

Table S3. Attributes description for the Cardiovascular Disease dataset

Attribute name	Attribute type	Mean (±std)	Categories (mode)
id	Numerical	$49972.41(\pm 28851.30)$	-
age (in days)	Numerical	$19468.86(\pm 2467.25)$	-
gender	Categorical	-	1,2 (1)
height	Numerical	$164.35(\pm 8.21)$	-
weight	Numerical	$74.2(\pm 14.39)$	-
ap_hi	Numerical	$128.81(\pm 154.01)$	-
ap_lo	Numerical	$96.63(\pm 188.47)$	-
cholesterol	Categorical	-	1,2,3 (1)
gluc	Categorical	-	1,2,3 (1)
smoke	Categorical	-	0,1 (0)
alco	Categorical	-	0,1 (0)
active	Categorical	-	0,1 (1)
cardio	Categorical	-	0,1 (0)

reasonably well, with PCD values below 0.21. Among the models, NPC, DP-NPC and GC were the only models to achieve no statistically significant difference ($p > 0.05$) correlation matrix compared to real data correlation matrix with PCD values between 0.0412 and 0.0115. NPC ($PCD = 0.0115$) and DP-NPC ($PCD = 0.0131$) exhibited the most visually similar matrices with the lowest PCD values. In contrast, TVAE, DP-TVAE, TabDif and DP-TabDif showed the least similarity visually, though their PCD values remained low (between 0.1572 and 0.2074). Apart from that, adding DP to the models did not visually impact correlation preservation quite much.

Figure S29 presents the mean DD plots across 10 folds, comparing the depth measurements of synthetic tabular data generated by each STDG model with those of real tabular data. Each plot includes the R^2 metric, which indicates the proportion of variance in the synthetic depths explained by real depths. All models except TVAE, DP-TVAE, TabDif and DP-TabDiff showed good depth alignment with R^2 values higher or around 0.8. The poorer fits were obtained with TVAE and DP-TVAE with a R^2 of 0. Overall, adding DP did not impact this metric for DP-TVAE. For the rest of the models (DP-NPC, DP-GC, DP-CTGAN and DP-TabDif), R^2 values were reduced compared to their non-DP counterpart.

Figure S30 illustrates the mean ROC curves with the AUC of the Random Forest classifier trained to differentiate between real and synthetic samples across 10 folds for each STDG model. CTGAN ($AUC - ROC = 0.7818$) and DP-CTGAN ($AUC - ROC = 0.7746$) were the only models closest to the ideal AUC-ROC of 0.5, indicating that the synthetic tabular data generated by these models was the least distinguishable from real data. In contrast, all other models showed higher AUC-ROC values, close to 1. Additionally, adding DP generally increased AUC-ROC score for most models, suggesting that DP makes synthetic samples more distinguishable from real ones.

Figure S31 summarises the fidelity results, displaying the mean, minimum, and maximum values across the 10 evaluation folds for each fidelity metric and STDG model, along with the associated p-value for comparison between model pairs (base and DP counterpart). This figure visually supplements Table 9 and

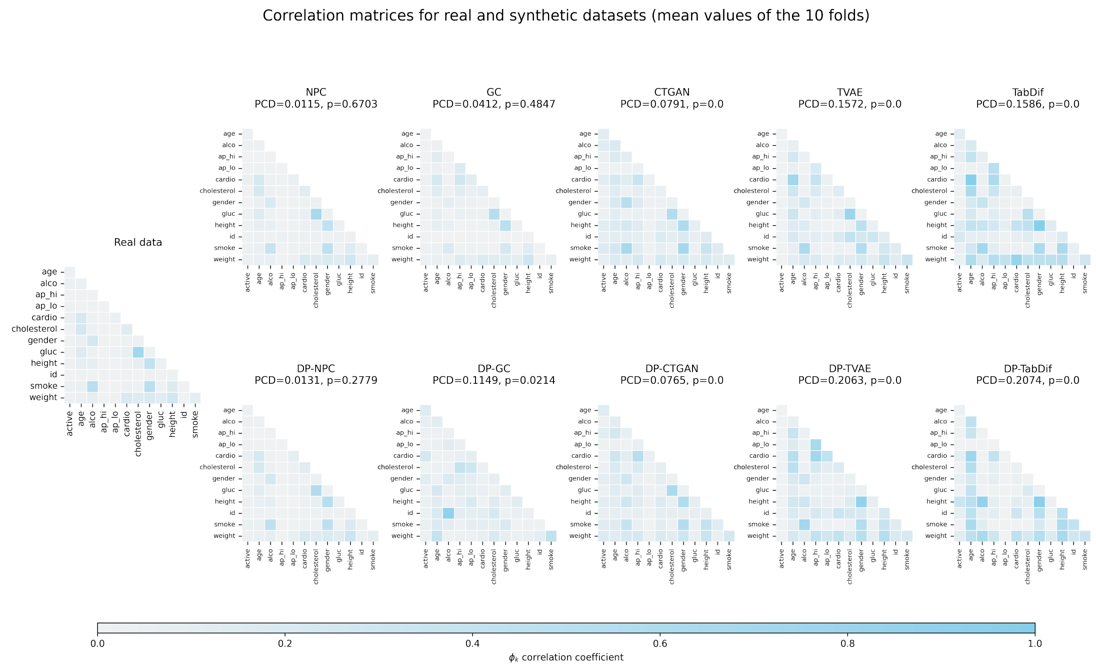

Figure S28. Correlation matrices of real data and synthetic data generated by each STDG model for Cardiovascular Disease dataset.

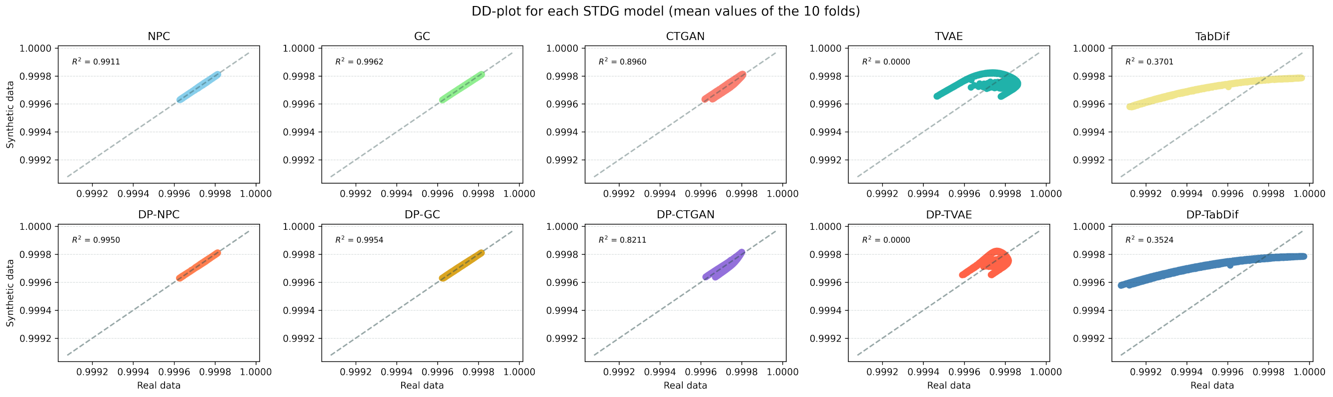

Figure S29. DDplot of real and synthetic data adjustments per each STDG model for Cardiovascular Disease dataset.

Section 3.3.1 of the main text, providing a clear representation of the variability and significance of fidelity metrics across models.

3.2 Utility results

Figure S32 illustrates the mean distributions of the classification metrics across the 10 folds for TRTR and TSTR with each STDG model. The plots include the associated p-values from a paired t-test comparing TRTR and TSTR distributions and the mean TRTR-TSTR metric differences (Δ) to highlight significant differences between classification metrics distributions. NPC, DP-NPC, GC, DP-TVAE, TabDif and DP-TabDif showed no statistically significant differences ($p > 0.05$) in classification metrics distributions compared to TRTR maintaing low classification metric differences ($\Delta < 0.14$). In contrast, DP-GC, CTGAN, DP-CTGAN and DP-TVAE showed statistically significant differences ($p < 0.05$) in classification

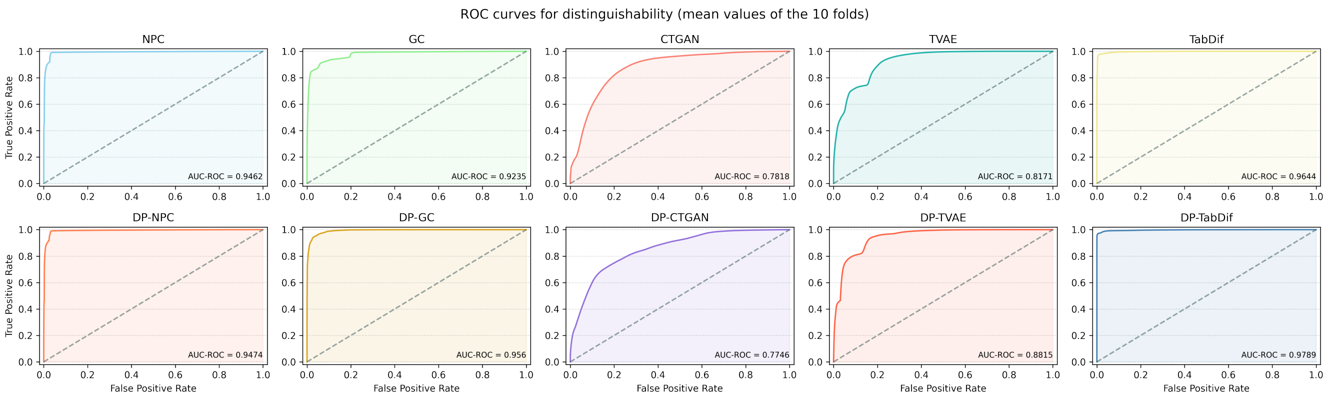

Figure S30. Distinguishability ROC curves of synthetic tabular data generated by each STDG model for Cardiovascular Disease dataset.

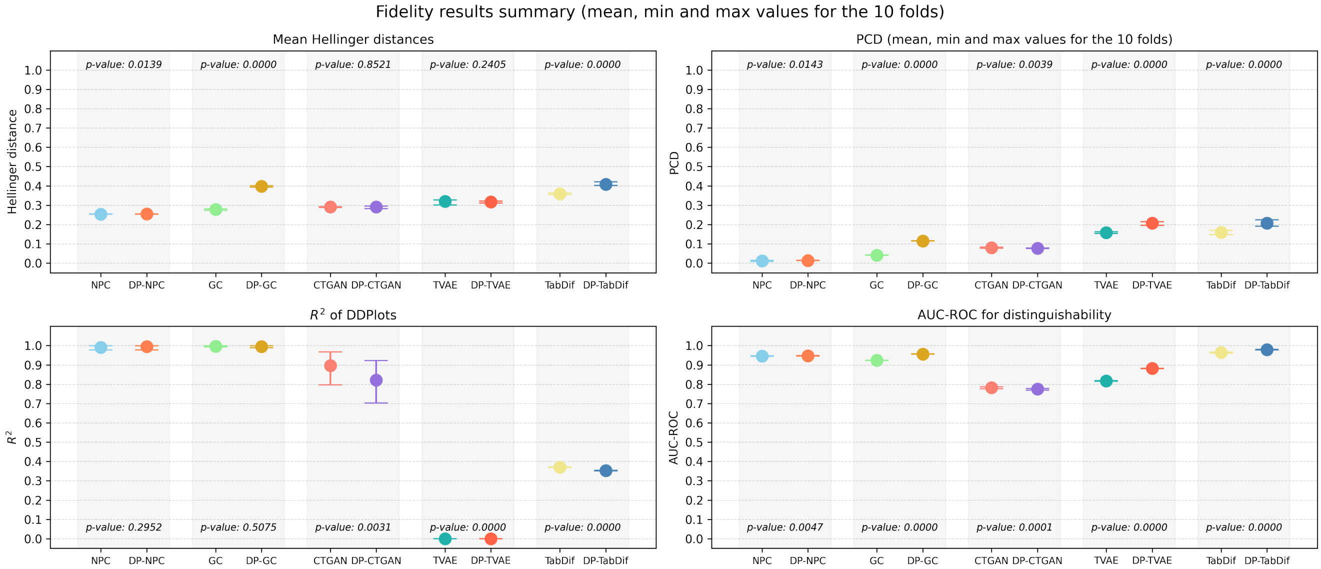

Figure S31. Fidelity results summary over the 10 folds for the synthetic tabular data generated by each STDG model for Cardiovascular Disease dataset.

metrics distributions compared to TRTR, but still maintained low classification metric differences ($\Delta <$ 0.12), being the higher ones for TVAE and DP-TabDif, ranging from 0.1009 to 0.1326. These results suggest that synthetic tabular data retains good utility for classification tasks across all models, despite all metrics distributions of TSTR for some models being significantly different compared to TRTR.

Figure S33 illustrates the classification metrics differences when TRTR and TSTR are computed, displaying the mean, minimum, and maximum metric differences across the 10 evaluation folds for each classification metric and STDG model, along with the associated p-value for comparison between model pairs (base and DP counterpart). This figure visually supplements Table 10 and Section 3.3.2 of the main text, providing a clear representation of the variability and significance of classification metrics differences across models.

Figure S34 illustrates the mean distributions of the regression metrics across the 10 folds for TRTR and TSTR with each STDG model. The plots include the associated p-values from a paired t-test comparing TRTR and TSTR distributions, as well as the mean TRTR-TSTR metric differences (Δ) to highlight

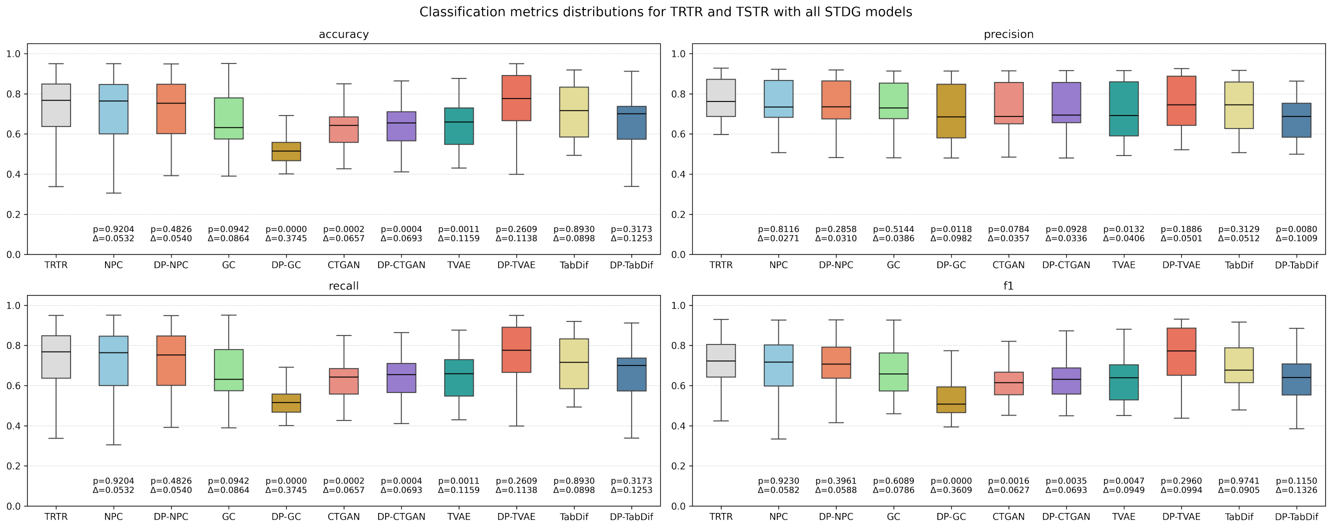

Figure S32. TRTR and TSTR results of classification metrics for Cardiovascular Disease dataset

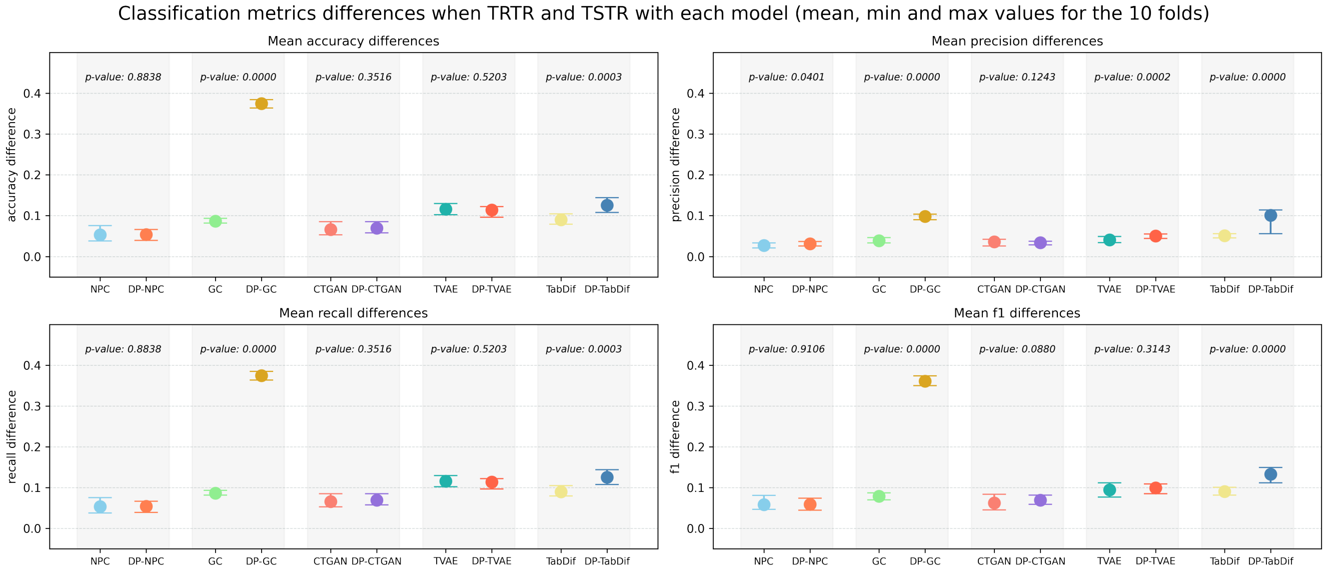

Figure S33. Classification differences between TRTR and TSTR results summay over the 10 folds for the synthetic tabular data generated by each STDG model for Cardiovascular Disease dataset.

significant differences between regression metrics distributions. All STDG models except NPC showed statistically significant differences ($p < 0.05$) in regression metrics distributions compared to TRTR, but still maintained low classification metric differences ($\Delta < 0.1$) for all models expect DP-TVAE, TabDif and DP-TabDif ranging from 0.1 to 0.6. These results suggest that synthetic tabular data retains good utility for regression tasks across all models except DP-TVAE, TabDif and DP-TabDif, despite all metrics distributions of TSTR for most models being significantly different compared to TRTR.

Figure S35 summarises the regression metrics differences when TRTR and TSTR, displaying the mean, minimum, and maximum metric differences across the 10 evaluation folds for each regression metric and STDG model, along with the associated p-value for comparison between model pairs (base and DP

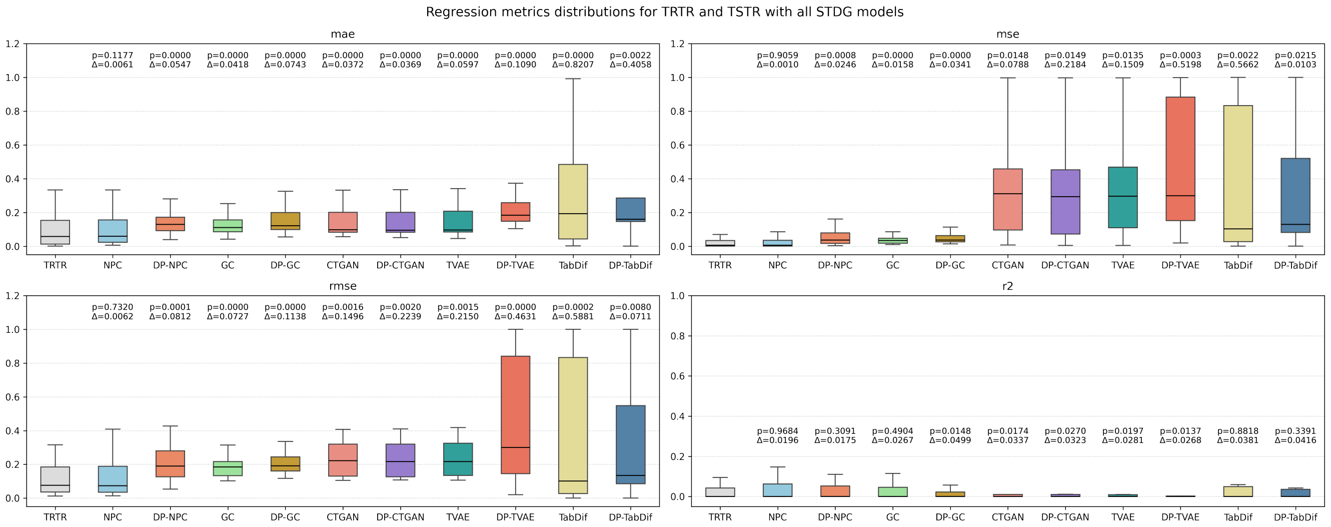

Figure S34. TRTR and TSTR results of regression metrics for Cardiovascular Disease dataset.

counterpart). This figure visually supplements Table 10 and Section 3.3.2 of the main text, providing a clear representation of the variability and significance of regression metrics differences across models.

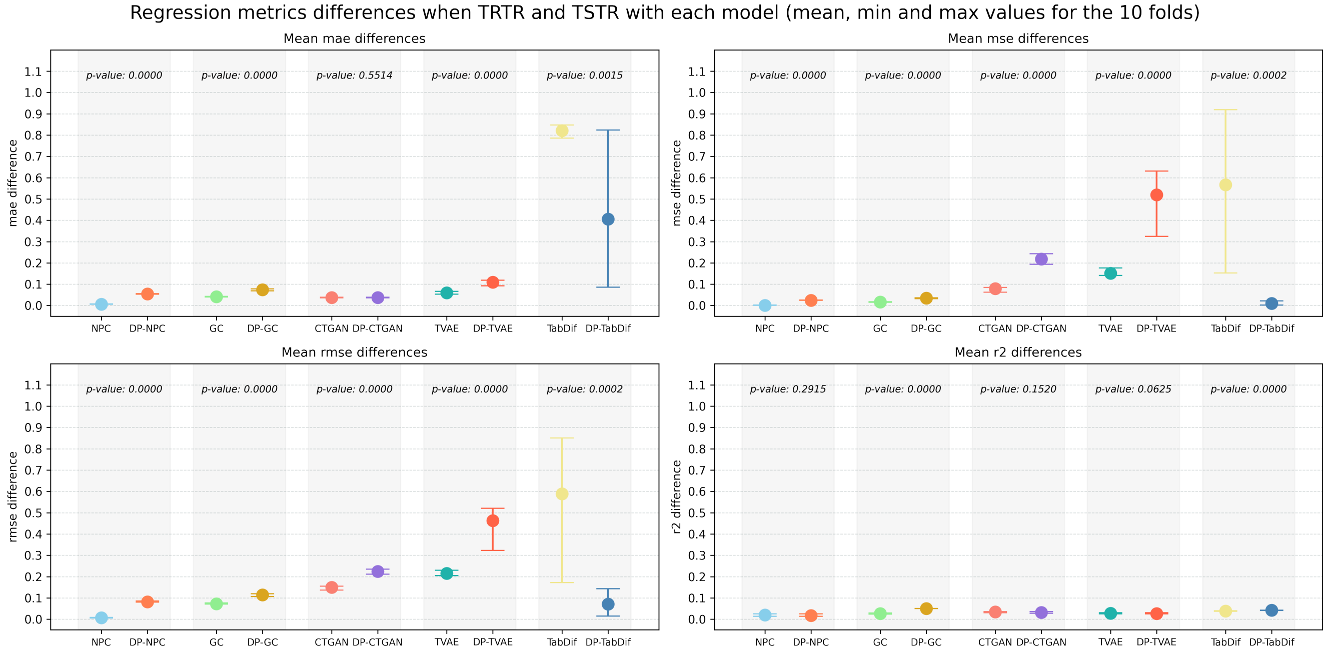

Figure S35. Regression differences between TRTR and TSTR results summay over the 10 folds for the synthetic tabular data generated by each STDG model for Cardiovascular Disease dataset

3.3 Tradeoff results

Figure S36 summarises the fidelity-utility tradeoff results, displaying the mean, minimum, and maximum values of the G metric across the 10 evaluation folds for each STDG model, along with the associated p-value for comparison between model pairs (base and DP counterpart). This figure visually supplements

tradeoff results of Table 9 and Section 3.3.1 of the main text, providing a clear representation of the variability and significance of the G metric across STDG models.

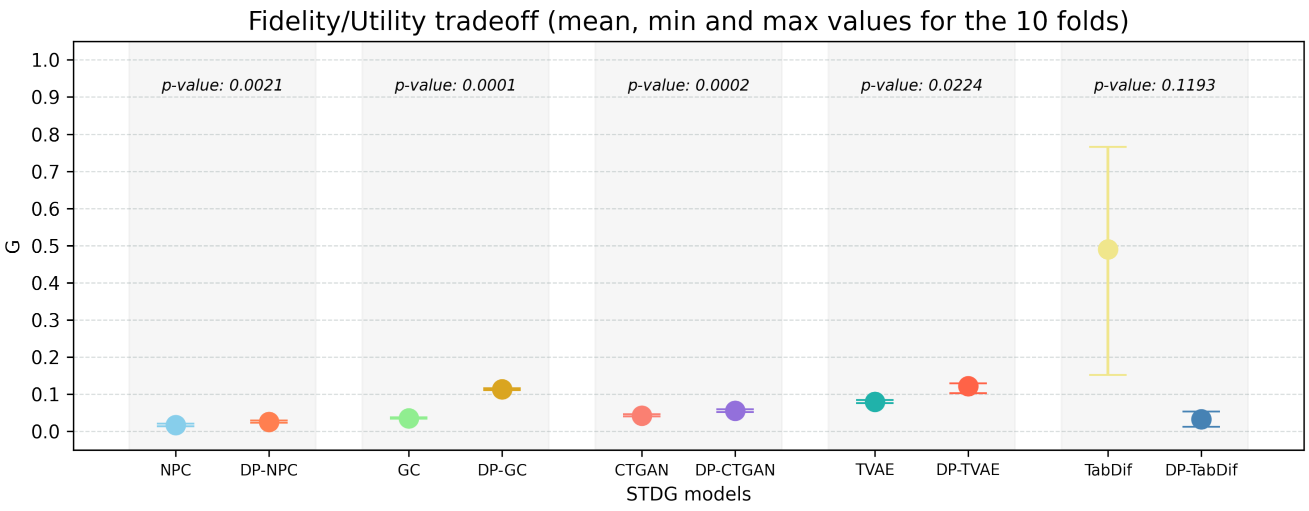

Figure S36. Tradeoff results summary over the 10 folds for the synthetic tabular data generated by each STDG model for Cardiovascular Disease dataset

3.4 Privacy attacks results

Figure S37 illustrates the attack risks with confidence intervals, averaged across the 10 synthetic tabular data folds for each STDG model and for linkability, univariate singlingout, multivariate singlingout and membership inference attacks. Linkability attack risks were near 0 across all models, while univariate and multivariate singlingout attack risks were very low (< 0.2) for all models. An exception was observed for the multivariate singlingout attack risks in TVAE (0.32) and DP-TVAE (0.35), which exceeded the risks observed from other models. Membership inference attack risks were highest for NPC (0.4), surpassing the risks observed for other models, which remained below 0.1. These results suggest that synthetic tabular data generated by all STDG models presents minimal privacy risks, except NPC, TVAE and DP-TVAE, which showed elevated risks in specific attacks.

Figure S38 presents the attributes inference attack risks with confidence intervals, averaged across the 10 synthetic tabular data folds for each STDG model and dataset attributes. The highest risks were observed for NPC and DP-NPC across all attributes, with risk values ranging from 0 to 0.4. In contrast, the risks for all other models remained below 0.1 for every attribute. These findings indicate that NPC and DP-NPC performed the worst in protecting against attribute inference attacks, with the attributes showing a likelihood of approximately 35% to be linked to real data attributes.

Figure S39 summarises the resulting risks for all privacy attacks, displaying the mean, minimum, and maximum risks across the 10 evaluation folds for each STDG model, along with the associated p-value for comparison between model pairs (base and DP counterpart). This figure visually supplements the privacy attack results of Table 11 and Section 3.3.3 of the main text, providing a clear representation of the variability and significance of the risks for privacy attacks across STDG models.

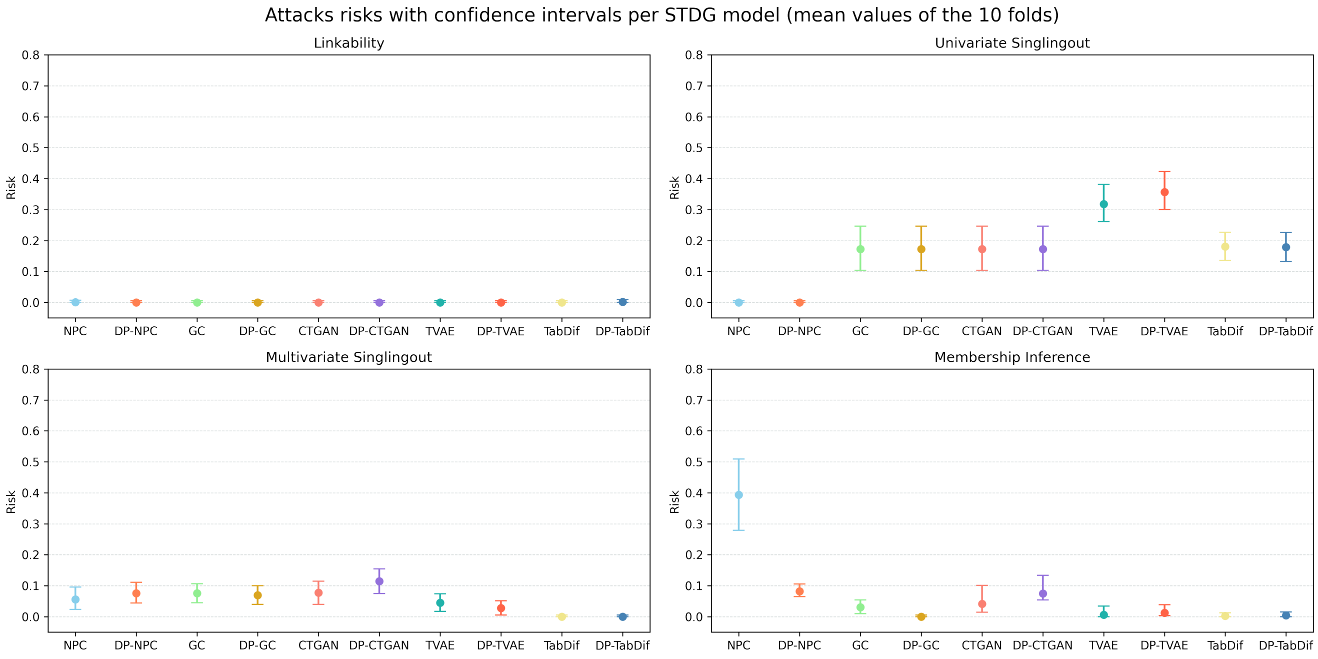

Figure S37. Attacks risks with confidence intervals for synthetic data generated by each STDG model for Cardiovascular Disease dataset.

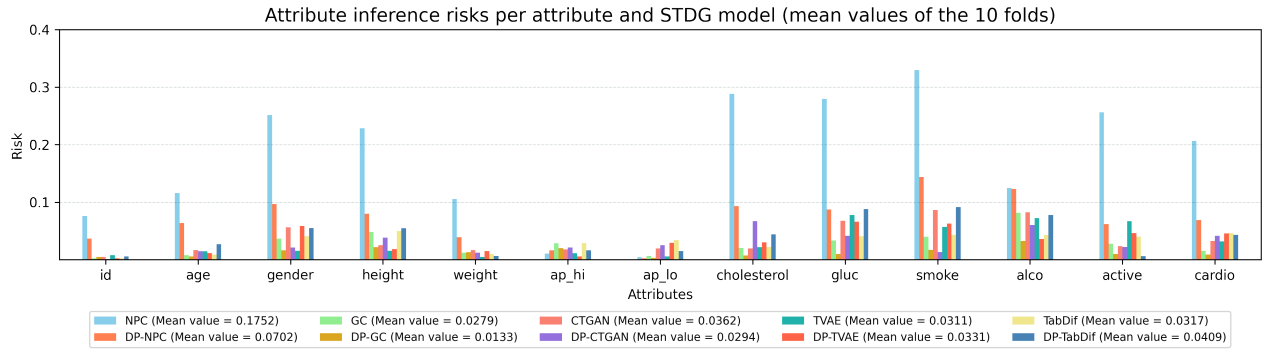

Figure S38. Attributes inference risks per attributes and STDG models for Cardiovascular Disease dataset.

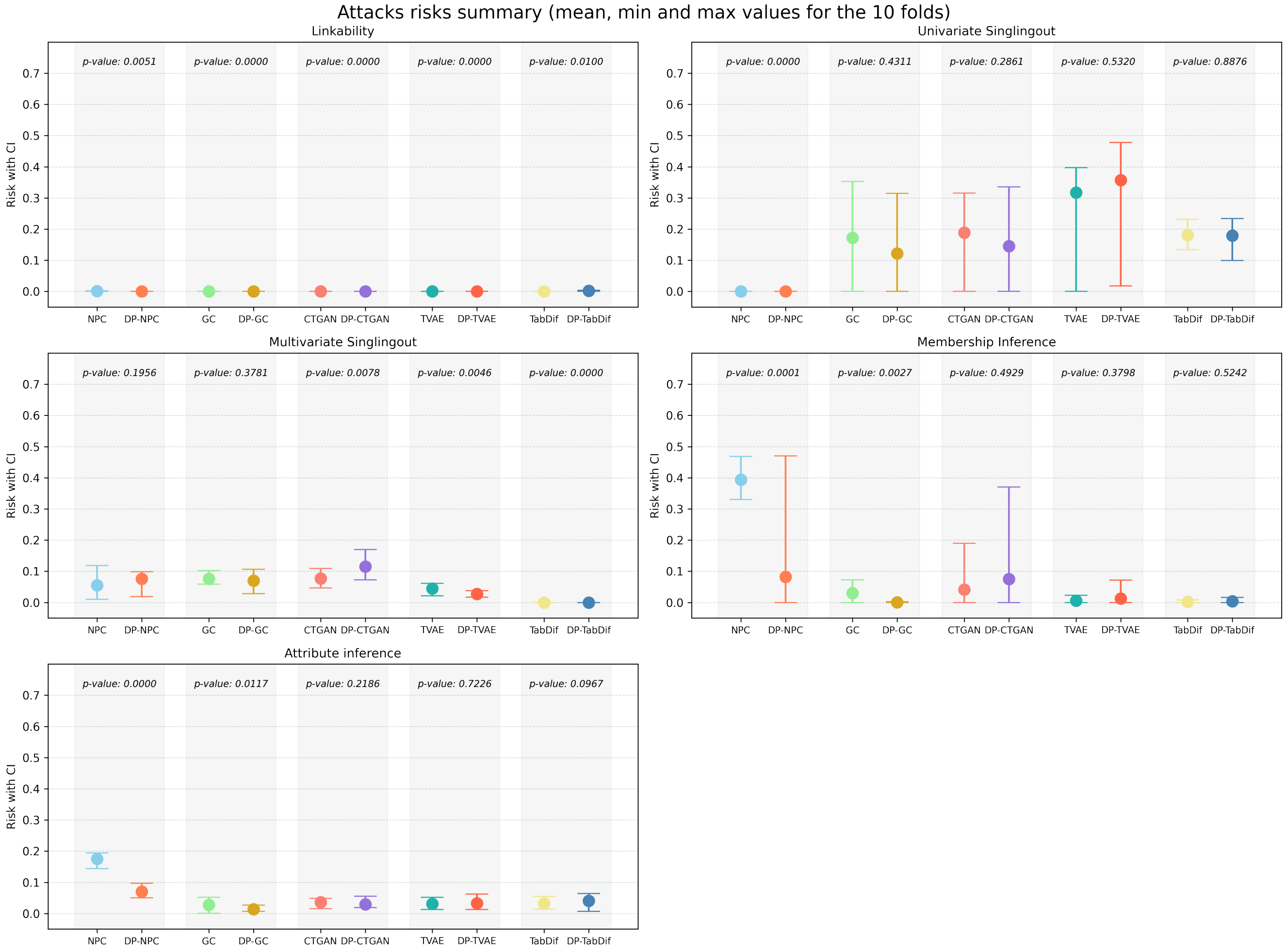

Figure S39. Attacks risks summary for synthetic data generated by each STDG model for Cardiovascular Disease dataset.
